# An Analysis of the Number of Medical Malpractice Claims and Their Amounts

**DOI:** 10.1371/journal.pone.0153362

**Published:** 2016-04-14

**Authors:** Marco Bonetti, Pasquale Cirillo, Paola Musile Tanzi, Elisabetta Trinchero

**Affiliations:** 1 Bocconi University and Carlo F. Dondena Centre for Research on Social Dynamics and Public Policy, Milan, Italy; 2 Applied Probability Group, Delft University of Technology, Delft, The Netherlands; 3 Research Division C. Demattè, SDA Bocconi School of Management, Milan, Italy; 4 Department of Economics, University of Perugia, Perugia, Italy; Azienda Ospedaliero-Universitaria Careggi, ITALY

## Abstract

Starting from an extensive database, pooling 9 years of data from the top three insurance brokers in Italy, and containing 38125 reported claims due to alleged cases of medical malpractice, we use an inhomogeneous Poisson process to model the number of medical malpractice claims in Italy. The intensity of the process is allowed to vary over time, and it depends on a set of covariates, like the size of the hospital, the medical department and the complexity of the medical operations performed. We choose the combination medical department by hospital as the unit of analysis. Together with the number of claims, we also model the associated amounts paid by insurance companies, using a two-stage regression model. In particular, we use logistic regression for the probability that a claim is closed with a zero payment, whereas, conditionally on the fact that an amount is strictly positive, we make use of lognormal regression to model it as a function of several covariates. The model produces estimates and forecasts that are relevant to both insurance companies and hospitals, for quality assurance, service improvement and cost reduction.

## Introduction

The subject of clinical risk management and patient safety is one of the main critical points in the supply of health services. Managing disputes or litigation—and the resulting impact on health care expenditure—is a priority both at the institutional and at the organizational level (see, e.g., [1, 2] and [3]).

Over the last few years, the growth and the aging in population, the rise in expectations in the levels of health, and the increasing ease of access to information have changed patients’ demands on health services, and increased the numbers of medical malpractice claims. In this paper, we focus our attention on the Italian case, where the growing financial restrictions placed on the Italian National Health Service, and the more pressing need for insurance companies to cover specific risks in the health sector are leading to changes in the basic risk management practices, and to the development of new local strategies. These trends, however, do not always appear to be based on a solid decisional process and seem, in a few cases, to be driven by short term considerations [4].

The Italian National Health Service, after several reforms, combines common central guidelines and decentralization of health policy responsibilities to the intermediate level of government. As well stated in [5], “the Central government has exclusive power to set system-wide rules and the health services that must be guaranteed throughout the country. Regions have responsibility for the organization and administration of publicly financed healthcare. Italian Regions differ widely in terms of demography, economic development (and fiscal capacity), health care infrastructures and health expenditures. (…) In the health sector Regions developed different organizational and funding models and now there are many relatively different regional health systems.”

Medical malpractice involves patient damage, injury or death attributed to negligent behavior by a medical practitioner or other health care professions [6]. Often patients (or their families), who think to have been victims of medical malpractice, file claims against health care providers. This possibility has a potentially strong impact in terms of costs and reimbursements, and it leads doctors, other health care professions and health care organizations to underwrite liability insurance policies in order to offset their risks.

Modeling claims due to alleged medical malpractice thus becomes very important from a legal, regulatory, and insurance point of view. A better understanding of such a phenomenon can have positive effects for hospitals and clinics in terms of quality assurance, service improvement, and cost reduction. At the same time, such understanding is essential for insurance companies to be able to reliably price their policies, in order to implement a more efficient risk management approach to losses, as required by new international regulations like Solvency II (see for example the discussion in [7]).

Notwithstanding the importance of the topic, the related statistical and actuarial literature is not extensive, as most contributions deal with the legal aspects and the impact on the medical profession (see [2], and references therein). This is probably due to the lack of publicly available data, as well as to the novelty of the phenomenon in many countries like Italy—the source of our data [8].

Some specific modeling contributions are discussed in [9–13], and [14]. In particular, the modeling approaches of [10] and [11] on US data have been a source of inspiration for some of the methods that we implement below.

Here we describe what is, to the best of our knowledge, the first published large analysis of the medical malpractice phenomenon in Italy, involving statistical models both for the number of claims and for their associated monetary amounts.

The main findings of our analyses, whose details are given in the rest of the paper, can be summarized as follows:

1. The inhomogeneous Poisson process is able to model the number of medical malpractice claims accurately. Its predicting power has been successfully back-tested.
2. In Italy, the yearly number of claims due to alleged Medical Malpractice has (linearly) increased over time in the last years. This is true for all the typologies of claims we have analyzed: injury, injury at birth, death, monetary damage and other. The regions of Toscana, Liguria and Lazio show the highest growth. Lombardia is the only region experiencing no particular trend in the number of claims.
3. The number of claims (for all possible types of causes, apart from monetary damage) is positively and significantly dependent on both the size of the hospitals and the complexity of the medical operations, as represented by the Case Mix Index (CMI) of the health care organization.
4. Importantly, a clear relationship between the number of claims and the type of medical departments involved in the analysis does not emerge.
5. Regarding the monetary amounts (corrected for inflation) that insurance companies have to pay in case of a successful claim, we observe an increase for claims related to the death of the patient, a stationary behavior for claims due to injuries at birth and monetary damages, and a slight decrease for non-birth injuries.
6. Differently from what we obtain for the number of claims, the type of medical department does have a significant effect on the monetary amounts. For example, Orthopedics and Obstetrics generate, on average, higher disbursement costs for hospitals and insurance companies.

In Section “The Data”, we describe the Italian medical malpractice claims data set that we have used for the analysis. In “Methods”, we summarize the statistical methodology that we have implemented to model the numbers of claims and the associated payout amounts. In “Results” we discuss the main findings, including point estimates, back-testing results, and forecasts. To avoid tens of tables, we do not include all the estimates and the forecasts produced as part of the research, but they are naturally available upon request to the authors. We close in the “Discussion” section with some summarizing comments and possible extensions of our work.

Three Appendices contain the statistical details and the complete descriptions of the models we have fitted.

## The Data

In this section we describe the data that we have used in our analyses and provide some basic descriptive information. The results of the in-depth analyses will be presented in the “Results” section.

As far as we know, the data set that we have used to study the problem of alleged medical malpractice represents the largest Italian data set of this type in the scientific literature. It has been obtained by pooling the data of three of the major international insurance brokers in Italy: AON, Marsh, and Willis Italy.

The observation window ranges from January 1st 2004 to December 31st 2012.

The data set contains a total of 38125 reported claims due to alleged cases of medical malpractice. These observations arise from 15 Italian regions (over a total of 20). From North to South: Valle D’Aosta, Veneto, Lombardia, Trentino-Alto Adige and Friuli-Venezia Giulia, Emilia-Romagna, Liguria, Toscana, Marche, Umbria, Lazio, Campania, Calabria, Puglia and Sicilia. Trentino-Alto Adige and Friuli-Venezia Giulia are two independent regions, but they are pooled together using the common classification *Nordest* (Northeast). The Italian regions that are not represented in our sample are: Piemonte, Abruzzo, Molise, Basilicata and Sardegna.

It is important to stress that regions, in Italy, refer only to an historical administrative partitioning of the territory, and, in this study, they were *not* constructed on the basis of the presence of any health care disparity.

The data set roughly contains 52% of all the hospitalizations in public hospitals in the available regions, with respect to 2012 data, that is 3,152,611 out of a total of 6,087,039. The best covered regions are Nordest and Lombardia, with a coverage of 100% and 83%, while the worst covered ones are Marche and Veneto, with 8% and 18% [15].

Regarding the representativeness of the sample, it is important to stress that the data have not been sampled randomly. This is due to the fact that our observations only come from those hospitals, which have underwritten an insurance contract with one of the three brokers, thus determining a selection bias.

For each claim the following information is available: Region, Hospital Code, Medical Department, Date of the Reporting of the Claim, Alleged Cause of the Claim. The claims can be due to injury (INJ), death (DEA), injury at birth (BIR), monetary damage to people and things (DAM) like a theft or a broken mobile, or to other causes (OTH). The need to disaggregate injuries at birth from the other injuries is due to the tremendous impact this type of events has, both from a personal and an insurance point of view. This disaggregation was suggested in one of the many discussions we had with practitioners and insurance brokers, when cleaning the data.

In addition, for each hospital, the total number of hospitalizations in 2012 is known, as well as the Case Mix Index (CMI). The CMI represents the complexity of a hospital’s patient mix (see [9, 16], and [17]). As such, we have used it as a measure of the average complexity of the procedures performed within each hospital.

We have classified the medical departments claims refer to as follows: Anesthesia (AN), Surgery (SU, all specializations apart from orthopedic surgery and emergency surgery), General Medicine (ME), Orthopedics (OR), Obstetrics and Gynecology (GY), Not Classifiable (NC), Health Support Services (HS, i.e. histology, laboratory, etc.), Emergency (ED), Other departments (OT), and Missing Information (NA). The NC category refers to the whole hospital: claims for the “NC department” are those claims that cannot be associated to any specific department within the given hospital/clinic. An example would be “falling from the stairs while hospitalized.” Note that this is not the same as “Other departments,” which indicates a separate group of known departments, for which only a small number of claims was recorded, thus suggesting the need of aggregation not to lose statistical significance. NC is also different from NA: while the first refers to the whole hospital, for the second we are just in a missing information situation (it could be surgery or anything else, but we do not know).

For many of the claims, the status of the claim (open or closed) is also known as detailed in Subsection “Amounts” below. For closed claims, the payoff amount, i.e. the payment settled by (or imposed to) the insurance company for that claim, is also available.

### Number of claims

Table 1 contains the number of claims by department and alleged cause of claim. From this table one can extract some interesting information. For example, it appears that most claims related to monetary damages are connected to the whole hospital (Not Classifiable, NC in our acronyms), where a mobile phone can be easily lost or stolen in the common areas, while injuries seem to be very often linked to surgery and orthopedics departments, probably because of the more invasive treatments.

**Table 1:** Claims by department and alleged cause (Jan. 1, 2004–Dec. 31, 2012).

Department	Other Cause (OTH)	Monetary Damage (DAM)	Death (DEA)	Injury at Birth (BIR)	Injury (INJ)	Total
Anesthesia (AN)	8	54	73	–	580	*715*
Surgery (SU)	264	583	690	–	6419	*7956*
General Medicine (ME)	73	886	591	–	2302	*3852*
Orthopedics (OR)	25	165	207	–	3826	*4223*
Obstetrics/Gynecology (GY)	86	47	251	717	1461	*2562*
Not Classifiable (NC)	314	1952	123	–	3069	*5458*
Health Support Services (HS)	18	112	49	–	1170	*1349*
Emergency (ED)	59	331	410	–	3351	*4151*
Other departments (OT)	889	458	173	–	1923	*3443*
Missing Information (NA)	1443	318	614	–	2041	*4416*
Total	*3179*	*4906*	*3181*	*717*	*26142*	**38125**

Anesthesia departments generate the smallest number of claims in the data set, probably because anesthesia is always coupled with some type of surgery, and the activities of this type of department are more visible to the patients. As expected, injuries or deaths at birth only concern the departments of obstetrics and gynecology.

**Fig 1:**
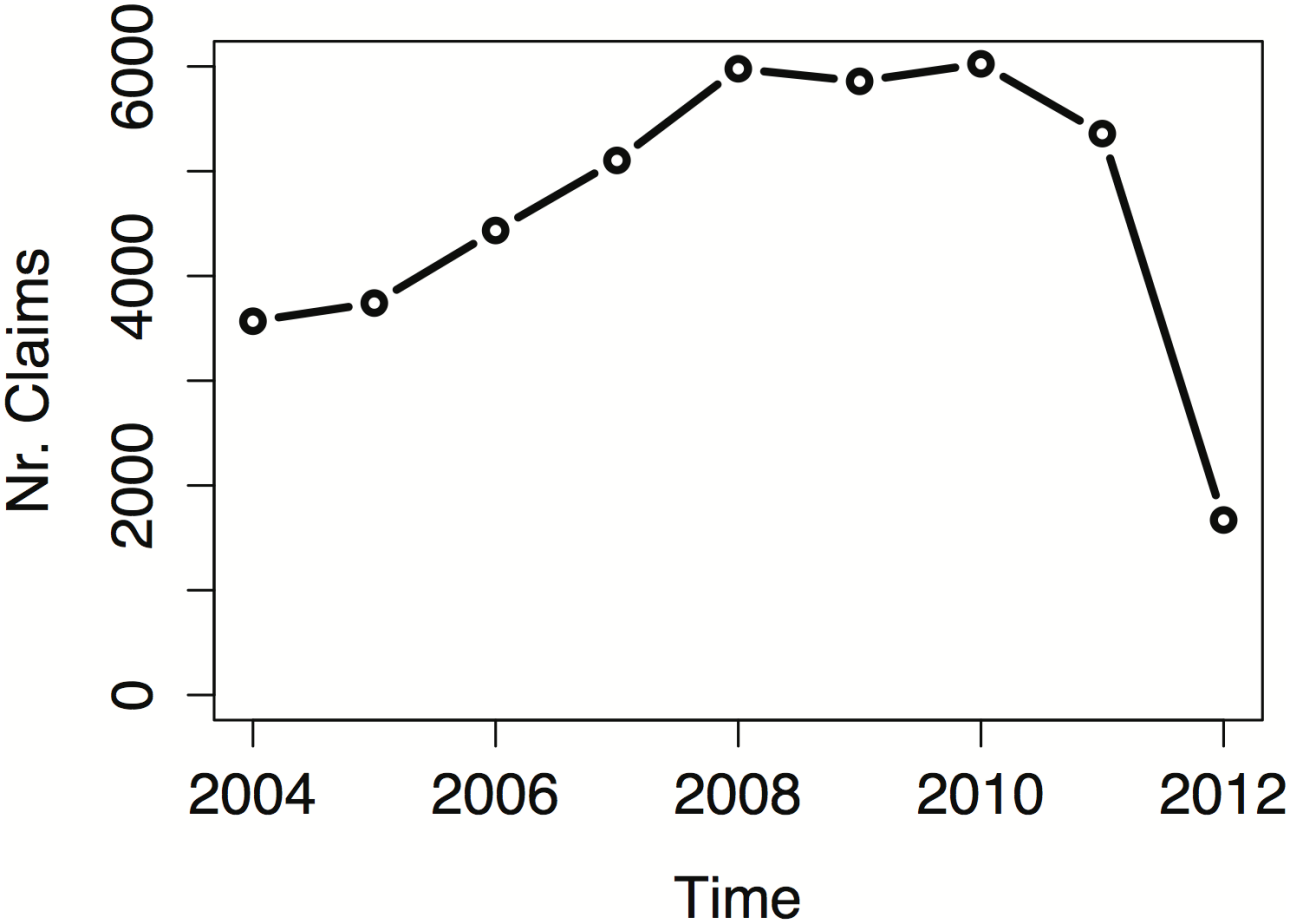
Total number of claims over time. Yearly number of claims for all regions pooled together (2004–2012).

Fig 1 shows the yearly number of reported claims, for all types of alleged causes, in the period 2004–2012. An overall increase in the number of reported claims is observed during the period 2004–2011, while we notice a drop in the number of reported claims in 2012. As a matter of the facts, at least one of the insurance companies was still collecting and organizing the data for the last months of 2012, so that those observations are not in our data set. To avoid the consequences of this recording delay, we have decided to restrict our attention on the 2004–2011 time window.

### Amounts

For the analysis of the payoff amounts, for which the recording delay is not as relevant, we have used a selection of the 38125 observations in the 2004–2012 time window, split among the different alleged causes of claim as shown in Table 2.

**Table 2:** Number of claims per alleged cause of claim, as used (Yes vs. No) in the analysis of the monetary amounts.

Used	Other Cause (OTH)	Monetary Damage (DAM)	Death (DEA)	Injury at Birth (BIR)	Injury (INJ)	Total
Yes	714	2966	806	239	11134	*15859*
No	2465	1940	2375	478	15008	*22266*
Total	*3179*	*4906*	*3181*	*717*	*26142*	*38125*

In particular, claims had status equal to Open (16971), Closed (14058), Without Further Action (WFA, 4574), or Unknown (2522). We have analyzed the amounts associated with claims having WFA (4574) or Closed statuses, and with a non missing amount (11285 out of 14058). We have corrected any missing amounts associated with WFA claims to be equal to zero. Claims with zero monetary amount but with Open status were removed from the analysis, as these were not true zeros being the claims still open. All 2522 claims with Unknown status also had missing amount, and were removed as well. All in all, a total of 15859 claims with monetary amount was therefore available for the analysis, as shown in Table 2. Table 3 shows the distribution of the claims used for the analysis of the amounts, by region and by type of department.

**Table 3:** Number of claims by department (columns) and geographic region (rows), as used in the analysis of the monetary amounts.

	OT	AN	SU	ME	NA	OR	GY	NC	HS	ED	Tot.
Calabria	4	1	9	1	54	0	4	17	1	3	*94*
Campania	0	0	1	0	12	0	0	7	0	0	*20*
Emilia-Romagna	6	31	203	111	0	147	54	148	41	151	*892*
Lazio	16	7	81	38	71	29	27	145	21	53	*488*
Liguria	545	109	669	272	0	464	137	349	117	332	*2994*
Lombardia	117	198	1644	948	3	834	469	962	214	891	*6280*
Marche	0	4	155	6	252	4	18	40	0	5	*484*
Nordest	666	15	339	262	5	180	98	182	47	180	*1974*
Puglia	1	0	0	0	49	0	1	3	0	0	*54*
Sicilia	27	10	53	22	0	36	30	97	7	16	*298*
Toscana	40	39	140	76	0	98	31	248	77	76	*825*
Umbria	58	3	85	19	446	13	16	105	5	16	*766*
Valle D’Aosta	4	10	29	15	0	32	11	9	12	21	*143*
Veneto	59	23	130	88	0	46	39	71	37	54	*547*
Total	*1543*	*450*	*3538*	*1858*	*892*	*1883*	*935*	*2383*	*579*	*1798*	**15859**

All amounts have been adjusted for inflation using the Consumer Price Index (CPI) elaborated by the Italian Institute of Statistics [18]. All amounts were converted into Jan 31, 2012 Euro levels by using a yearly (geometric) average CPI of 2.15%.

The median payment was equal to 984 euros, the average to 26,220 euros, and the observed maximum to 5,387,470 euros. Table 4 shows the maximum monetary amounts observed within each combination of department by type of claim.

**Table 4:** Largest observed monetary amounts by alleged cause of claim and department.

Department	Injury at Birth (BIR)	Other Cause (OTH)	Monetary Damage (DAM)	Death (DEA)	Injury (INJ)
*Number of Claims*	*239*	*714*	*2966*	*806*	*11134*
Other departments (OT)	0	17108	14106	180322	833516
Anesthesia (AN)	–	NA	16178	492278	1053276
Surgery (SU)	–	99734	65882	1365691	2106096
General Medicine (ME)	–	18676	28416	1350717	5387470
Missing Information (NA)	–	18503	9563	1050490	1018125
Orthopedics (OR)	–	408797	5636	1086929	2232578
Obstetrics/Gynecology (GY)	4721106	29386	176624	1180281	2667626
Not Classifiable (NC)	–	42908	518510	410159	275546
Health Support Services (HS)	–	8723	16733	773564	714259
Emergency (ED)	–	1521	158378	1011962	1258203
All	4721106	408797	518510	1365691	5387470

**Fig 2:**
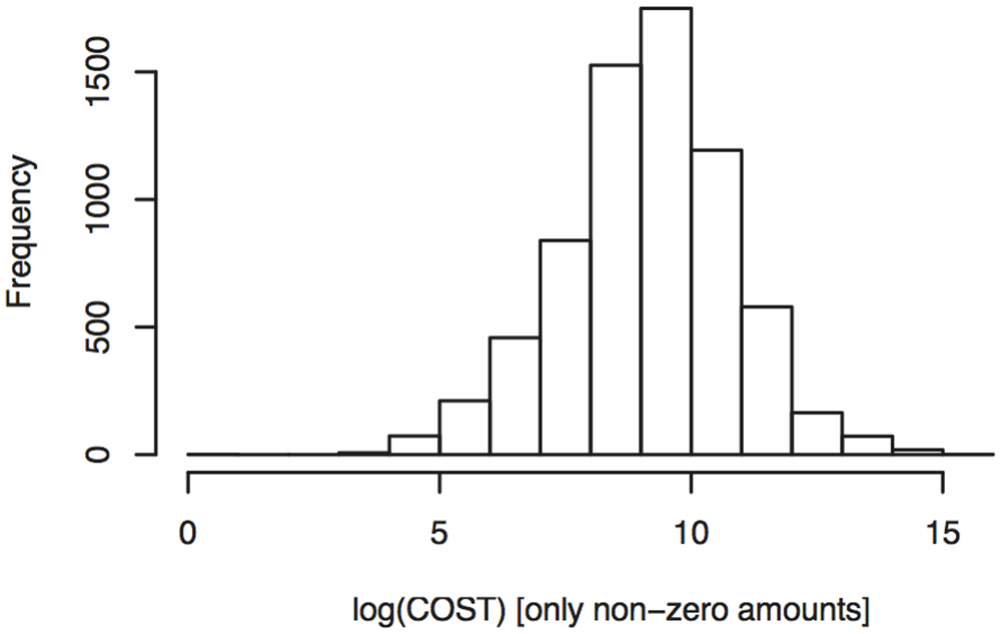
Histogram of non-zero payments. Histogram of log-transformed non-zero payments related to injuries.

A preliminary analysis of the claim amounts, all together and by type of claim, suggested a marginal lognormal model for the non-zero payments. As an example, Fig 2 shows the histogram of log-transformed non-zero payments for claims related to injuries. In Appendix 1 we describe additional analyses that further support the use of the lognormal distribution in our analyses of the amounts.

For the open claims, information about the amounts reserved by the insurance companies was sometimes also available, and we did indeed repeat all the analyses using that information as well. For brevity, here we do not include those additional analyses, but they are available upon request.

## Methods

In this section we summarize the modeling approach that we have followed. The technical details of such approach are described in Appendix 2.

### Modeling the number of claims

For modeling the numbers of claims we have used an inhomogeneous Poisson process, choosing the combination medical department by hospital as the unit of analysis. This means that all claims are gathered according to such combinations; in other words, each medical department by hospital unit is treated as a separate generator of claims. This is different from what Cooil [10] and Gibbons et al. [11] did in their works, where the unit of analysis was the single physician.

For each unit of analysis *i*, *i* = 1, …, *m*, with *m* the number of units, we modeled the number of claims by an inhomogeneous Poisson process whose time-varying intensity function is linearly dependent on a set of covariates (including time itself).

In the analysis we used the following covariates:

*x*_*i*,1_: the CMI of the hospital that the unit of analysis *i* belongs to. This is used as a measure of the complexity of the medical services offered by the hospital;
*x*_*i*,2_: the total number of hospitalizations (HOS) in 2012 for the hospital the unit of analysis *i* belongs to. This quantity represents a proxy for size. Given the lack of more precise information—we have assumed that the size of each hospital in 2012 could also describe its size for the previous years (and speaking with sanitary experts this appears to be a reasonable assumption on a short time scale);
*x*_*i*, *j*_: for *j* = 3, …, 10, a set of 8 dichotomic variables used to identify the different types of medical departments:
  *x*_*i*,3_: Department of Anesthesia, AN
  *x*_*i*,4_: Dept. of Surgery (all specializations except orthopedic surgery and emergency surgery), SU
  *x*_*i*,5_: Dept. of General Medicine, ME
  *x*_*i*,6_: Dept. of Orthopedics, OR
  *x*_*i*,7_: Dept. of Obstetrics and Gynecology, GY
  *x*_*i*,8_: Not Classifiable, NC
  *x*_*i*,9_: Health Support Services (e.g. labs), HS
  *x*_*i*,10_: Emergency Department, ED.
  The departments “OT” (Others), which contains all the non-specified departments, and “NA”, missing information, are treated as residual, and they are thus incorporated into the intercept *x*_*i*,0_ (INT), in order to avoid collinearity. For the number of claims, we are indeed interested in identifying departmental effects for major departments only.

For each reported claim, the date of the event and the date of reporting are available. After consultation with the brokers who provided the data we have decided to work with the latter only, i.e. with the time when a claim first appears in the database (clearly the date of an event appears in the database only after reporting has occurred). This decision was based on the fact that the reporting date is what really matters for insurance-related considerations. One should in fact expect some delay in the reporting of claims. Comparing the date of the reported claim with the date of the event generating it, we have found out that the overall average delay is equal to 1.69 years. Claims due to monetary damages are typically reported after 72 days from the event, while claims due to injuries at birth are reported on average after 742 days.

Consistently with such consideration, no adjustment has been performed for departments that were added or removed from the set of the claim-generating process over the years. As a consequence, such changes are reflected into the brokers’ databases as changes in the intensity of the reporting process.

The model was initially estimated at the national level. However, the Italian National Health Service allows the different regions to have diverse regimes of health governance, provided that a minimum level of service quality is guaranteed. As a consequence it seems more reasonable to estimate the model separately for each of the available regions, rather than just using one single model with intercept modifiers for the distinct regions.

As mentioned above, claims were grouped into five macro-sets (types of claims): claims due to injuries (injuries, INJ), claims due to injuries at birth (Birth, BIR), claims due to death (Death, DEA), claims due to monetary damages to people and things (Damages, DAM), and claims falling into other categories (Other, OTH).

We have estimated a total of 23 models, that is one for each of the (75) combinations of regions by types of claims for which a sufficient number of observations were available (including the case pooling together all observations, without regional differences, which we call “ALL”.).

The estimation of the models was performed using maximum likelihood. It is worth pointing out that the model for the claims due to injuries and deaths at birth is different, since these claims can only arise from departments of obstetrics and gynecology.

### Modeling the amounts

#### The two-component model

The inflation-adjusted liquidation cost/payment (*C*) has been modeled separately for the zero and the non-zero amounts, using a two-step regression approach:

1. A logistic regression model for the probability that a claim is closed with a zero payment.
2. Conditionally on an amount being strictly positive, a lognormal regression model for the amount *C*.

Both regression models have been developed to assess the statistical significance of the different regions, of the medical departments and of time (allowing for a possible quadratic effect of time on the two outcomes as well). It is worth underlining that the two (distinct) model selection processes will in general produce *different* sets of significant covariates. As a consequence, some care must be used to properly keep track of this fact in the later production of forecasts for the costs. In Appendix 2.2 we provide more details, and we also explain how to obtain the prediction intervals for the conditional expected values of (positive) costs, as well as for the *overall* mean costs.

#### Expected costs and tail amounts

Under the assumption that the expected values of the costs do not depend on their number, the expected value of the overall amount for a given time interval can be estimated as the product of the expected number of events and the expected amount for each event. Hence such average total amount can be easily computed from the models for the number of claims and the associated amounts (more details in Appendix 2.2).

One could also study the distribution of the total (regional or national) amounts, and in particular the quantiles of such distributions (the well-known Value at Risk—*VaR*—approach in risk management). This study would require an extensive simulation study from the joint distribution of the number of events and their amounts, and for completeness it should also take into account the sampling variability of the estimated parameters of the models. Such an approach would however still produce strongly model-dependent total amounts. As a matter of fact, the goodness of fit of the models, for the largest total amounts, would probably be very hard to assess, and the exercise could lead to a dangerous over-interpretation of the evidence contained in the data.

On the other hand, some information on such high-amount claims is indeed desirable. In Appendix 2.3 we describe how one can study the probabilities that some of the predicted total numbers of events lie in the extreme tail of the amount distribution, and we provide details on how to estimate their average value, the “Expected Shortfall”.

## Results

In this section we describe the main results from the analyses. For all the remaining cases, we are available to share them upon request.

### Number of claims

Starting from the original 38125 claims, restricting our attention to the period 2004–2011 and imposing the condition of having a value for all covariates of interests, we have analyzed 36981 observations.

Claims have been grouped into five macro-classes: INJ, DEA, DAM, OTH and BIR. We will discuss the first four in the next paragraph, and the BIR data in Subsection “Number of claims for injuries at birth”. This separation is due to our modeling choices, as explained in Appendix 2.1.

Remember that, in what follows, when using the dummy variables for the different departments, the intercept contains both OT and NA (defining the residual OT/NA group).

#### Number of non-birth-related claims

Within each class we have estimated models for all the data pooled together (i.e. without regional distinctions), and models for each region for which a sufficient number of observations were available. The model parameters were estimated on all 2004–2011 data. Model selection has then been performed using the Akaike’s information criterion (AIC), as common in these cases [10]. Tables 5, 6, 7, 8 and 9 contain some examples of the results for injuries (all regions, Lombardia and Toscana), deaths (Lombardia), and monetary damages (Liguria).

**Table 5:** Results for injuries, for all data pooled together (ALL). Estimates of the parameters of the Poisson model as per Appendix 2.1, number of departments of a given type that generated each alleged type of claim (N.Dep), observed frequencies of claims for the different types of departments (Obs.F), expected frequencies according to the model (Exp.F), and predicted claims for 2012 (P2012) and 2013 (P2013), together with their standard deviations (in brackets).

*δ*^⋆^	INT	CMI	HOS	AN	SU	ME	OR	GY	NC	HS	ED
1.70*	1.31*	0.25*	0.03*	-1.41*	0.73*	-0.27^+^	0.27*	-0.66*	-	-0.88*	0.17*
Dept. Type					N.Dep	Obs.F	Exp.F	P2012		P2013	
Baseline (OT/NA/NC)					176	5364	5365	730 (27.0)		976 (31.24)	
Anesthesia (AN)					70	539	539	79 (8.9)		92 (9.6)	
Surgery (SU)					87	5590	5590	735 (27.4)		1024 (32.0)	
General Medicine (ME)					84	1999	1999	284 (16.9)		351 (18.7)	
Orthopedics (OR)					83	3292	3292	421 (20.5)		626 (25.0)	
Obstetrics/Gynecology (GY)					79	1260	1260	180 (13.4)		221 (14.9)	
Health Support Services (HS)					83	1069	1069	162 (12.7)		178 (13.3)	
Emergency (ED)					80	2899	2899	426 (20.6)		496 (22.3)	

One asterisk * indicates significance at the 5% level, the plus + at 1%. Only for *δ*, the star ⋆ indicates that the estimate is also significantly different from 1 at the 5% level (*δ* = 1 corresponds to no trend in the model described in Appendix 2.1).

**Table 6:** Results for injuries, for the Lombardia region. Estimates of the parameters of the Poisson model as per Appendix 2.1, number of departments of a given type that generated each alleged type of claim (N.Dep), observed frequencies of claims for the different types of departments (Obs.F), expected frequencies according to the model (Exp.F), and predicted claims for 2012 (P2012) and 2013 (P2013), together with their standard deviations (in brackets).

*δ*	INT	CMI	HOS	AN	SU	ME	OR	GY	NC	HS	ED
0.96*	0.34*	0.11*	0.51*	-	2.47*	1.68*	1.99*	1.26*	1.45*	0.67*	2.02*
Dept. Type					N.Dep	Obs.F	Exp.F	P2012		P2013	
Baseline (OT/NA/AN)					46	385	389	45 (6.7)		63 (7.8)	
Surgery (SU)					25	2338	2338	305 (17.5)		331 (18.2)	
General Medicine (ME)					25	1062	1062	138 (11.7)		151 (12.3)	
Orthopedics (OR)					24	1377	1377	156 (12.6)		218 (14.7)	
Obstetrics/Gynecology (GY)					24	674	674	87 (9.3)		96 (9.8)	
Not Classifiable (NC)					25	842	842	106 (10.3)		123 (11.1)	
Health Support Services (HS)					25	386	386	42 (6.5)		63 (7.8)	
Emergency (ED)					24	1427	1427	183 (13.5)		205 (14.3)	

One asterisk * indicates significance at the 5% level, the plus + at 1%. Only for *δ*, the star ⋆ indicates that the estimate is also significantly different from 1 at the 5% level (*δ* = 1 corresponds to no trend in the model described in Appendix 2.1).

**Table 7:** Results for injuries, for the Toscana region. Estimates of the parameters of the Poisson model as per Appendix 2.1, number of departments of a given type that generated each alleged type of claim (N.Dep), observed frequencies of claims for the different types of departments (Obs.F), expected frequencies according to the model (Exp.F), and predicted claims for 2012 (P2012) and 2013 (P2013), together with their standard deviations (in brackets).

*δ*^⋆^	INT	CMI	HOS	AN	SU	ME	OR	GY	NC	HS	ED
1.61*	1.12*	-0.54*	2.75*	-0.56*	0.89*	0.22*	0.51*	-0.54*	0.60*	-	0.39*
Dept. Type					N.Dep	Obs.F	Exp.F	P2012		P2013	
Baseline (OT/NA/HS)					19	346	348	103 (10.1)		146 (12.1)	
Anestesia (AN)					8	82	82	25 (5.0)		34 (5.8)	
Surgery (SU)					11	419	416	134 (11.6)		168 (13.0)	
General Medicine (ME)					11	169	169	58 (7.6)		64 (8.0)	
Orthopedics (OR)					10	285	285	94 (9.7)		112 (106)	
Obstetrics/Gynecology (GY)					9	95	95	29 (5.4)		40 (6.3)	
Not Classifiable (NC)					11	316	316	117 (10.8)		171 (13.1)	
Emergency (ED)					11	254	254	87 (9.3)		97 (9.8)	

One asterisk * indicates significance at the 5% level, the plus + at 1%. Only for *δ*, the star ⋆ indicates that the estimate is also significantly different from 1 at the 5% level (*δ* = 1 corresponds to no trend in the model described in Appendix 2.1).

**Table 8:** Results for deaths, for the Lombardia region. Estimates of the parameters of the Poisson model as per Appendix 2.1, number of departments of a given type that generated each alleged type of claim (N.Dep), observed frequencies of claims for the different types of departments (Obs.F), expected frequencies according to the model (Exp.F), and predicted claims for 2012 (P2012) and 2013 (P2013), together with their standard deviations (in brackets).

*δ*	INT	CMI	HOS	AN	SU	ME	OR	GY	NC	HS	ED
0.93*	-1.33*	0.11*	0.66*	0.05*	1.80*	1.98*	0.72*	0.67*	-	0.11*	1.59*
Dept. Type					N.Dep	Obs.F	Exp.F	P2012		P2013	
Baseline (OT/NA/NC)					26	40	40	4 (2.0)		7 (2.6)	
Anestesia (AN)					14	20	21	2 (1.4)		4 (2.0)	
Surgery (SU)					24	203	203	25 (5.1)		28 (5.3)	
General Medicine (ME)					25	250	250	29 (5.4)		37 (6.1)	
Orthopedics (OR)					19	55	55	6 (2.4)		8 (2.8)	
Obstetrics/Gynecology (GY)					21	57	57	7 (82.6)		8 (2.8)	
Health Support Services (HS)					16	26	25	3 (1.7)		4 (2.0)	
Emergency (ED)					23	157	157	17 (4.1)		24 (4.9)	

One asterisk * indicates significance at the 5% level, the plus + at 1%. Only for *δ*, the star ⋆ indicates that the estimate is also significantly different from 1 at the 5% level (*δ* = 1 corresponds to no trend in the model described in Appendix 2.1).

**Table 9:** Results for monetary damages, for the Liguria region. Estimates of the parameters of the Poisson model as per Appendix 2.1, number of departments of a given type that generated each alleged type of claim (N.Dep), observed frequencies of claims for the different types of departments (Obs.F), expected frequencies according to the model (Exp.F), and predicted claims for 2012 (P2012) and 2013 (P2013), together with their standard deviations (in brackets).

*δ*^⋆^	INT	CMI	HOS	AN	SU	ME	OR	GY	NC	HS	ED
1.52*	-0.92*	-0.18*	1.88*	-0.10*	-	1.30*	-	-	2.43*	-	-
Dept. Type					N.Dep	Obs.F	Exp.F	P2012		P2013	
Baseline (OT/NA/SU/OR/GY/HS/ED)					21	137	139	19 (4.4)		23 (4.6)	
Anestesia (AN)					3	8	8	1 (1.0)		0 (0.0)	
General Medicine (ME)					8	88	89	5 (2.2)		7 (2.6)	
Not Classifiable (NC)					9	282	283	3 (1.7)		4 (2.0)	

One asterisk * indicates significance at the 5% level, the plus + at 1%. Only for *δ*, the star ⋆ indicates that the estimate is also significantly different from 1 at the 5% level (*δ* = 1 corresponds to no trend in the model described in Appendix 2.1).

A first consideration from Tables 5 to 9 is that the inhomogenous Poisson process correctly replicates the observed numbers of claims. Indeed, the maximum difference between observed and fitted numbers of claims, among all models, is an overestimation by 4 units.

Each table also contains predictions for years 2012 and 2013, on the basis of the models estimated up to the end of 2011. It will be interesting to verify them with actual data, should they become available to us.

For what concerns the estimates of the parameters of the model, it is worth noticing that most of them are significant at the 5% level of significance. For example, in Table 5, where we consider the claims due to injuries in all regions pooled together, all parameters are significantly different from zero apart from the one related to NC, the Not Classifiable category. Thus, when analyzing all claims for injuries without any regional distinction, the NC “departments” show no particular difference with respect to the baseline. In other words, after model selection, NC “departments” are included within the new OT/NA/NC group.

As expected, the size of the hospitals (in terms of patients in 2012) and the complexity of the operations (as expressed by the CMI) have, on average, a positive influence on the expected number of claims, especially for what concerns claims due to injuries and deaths. For what concerns claims due to monetary damages, conversely, it is not possible to obtain a clear relation with respect to CMI, but this is in line with the nature of the claims, not really related to the complexity of hospital operations; while the size of the hospital has a positive effect: the larger the number of patients, the larger—on average—the number of small economic losses.

For what concerns the dummy variables representing the departments, it is not possible to identify a unique behavior. This is quite surprisingly, since one would for example expect surgery departments to be riskier than the average.

We should note that the parameter *δ* is always strictly larger than 0, most of the times larger than 1 as well, but smaller than 2. In our model (Eq (6) in the Appendix), this means that an underlying linear trend is enough to model the average increase in the number of claims over time (see also Fig 1). An increase in the number of claims is present in all regions, with the only exception of Lombardia region, where no significant trend is observed (in Table 6, for instance, for claims due to injuries in Lombardia, *δ* can be safely constrained to 1).

In order to assess the predictive power of the model we have performed some back-testing experiments. In particular, we have estimated the model parameters using data until December 31st 2010, and have used the estimates to predict the number of claims in 2011. Predictions were then compared to the observed numbers of claims in 2011 for the different alleged claim causes, department types, and regions.

The results were quite satisfactory. For example, Table 10 shows the comparison for the numbers of claims due to injuries (INJ) using data from Lombardia region. The worst prediction in the table is obtained for the department of general medicine (ME): the actual number of claims is 122 while the model predicts 145 claims, with an error of 18.8%. The best prediction is given for gynecology and obstetrics, where the error is just 1%. In general, the most problematic units are the departments of general medicine (ME) and the Not Classifiable (NC) ones. The maximum error is equal to 19.7% for the claims due to injuries, in the general medicine departments in Tuscany. The prediction error across all cases is around 12%.

**Table 10:** Example of backtesting for claims due to alleged injuries for the Lombardia region. Observed (historical) claims against claims predicted for 2011.

Department	OT/NA/NC	SU	ME	OR	GY	NC	HS	ED
Observed	24	292	122	152	91	89	49	170
Predicted	25	314	145	177	92	105	53	185
Absolute Error	1	22	23	25	1	16	4	15

#### Number of claims for injuries at birth

The model for the number of claims due to injuries and deaths at birth is different from the one given in Eq (6) in the Appendix. In particular, we no longer need the covariates *x*_*i*,3_, ⋯, *x*_*i*,10_, given that all claims belong to the same department: Obstetrics and Gynecology. The data set contains 717 claims due to injuries and deaths at birth (go back to Table 1). These claims mainly come from Lombardia, Emilia-Romagna, Liguria, Toscana, Lazio and Calabria. For the other regions the number of observations is not sufficient to estimate the model reliably.

Table 11 contains the estimates of the parameters of the model, the predicted claims in 2012 and 2013 and their standard deviations, for all the claims pooled together (ALL), and for the different regions for which the model is estimable. The number of hospitalizations appears to be the most important covariate, while CMI is significant only for the pooled data and for the Lazio region. As usual model selection has been performed using AIC.

**Table 11:** Estimates and predicted claims due to injuries at birth. The asterisk indicates significance at 5% level, the star ⋆ indicates that *δ* is also significantly different from 1 at the 5% level. In brackets, we provide the standard deviations of the predicted claims in 2012 (P2012) and 2013 (P2013).

Region	*δ*	INT	CMI	HOS	P2012	P2013
ALL	1.21*^⋆^	-	0.26*	-	118 (10.9)	133 (11.5)
Calabria	1.94*^⋆^	-	-	1.52*	9 (3.0)	13 (3.6)
Emilia-Romagna	5.00*^⋆^	-6.24*	-	1.38*	11 (3.3)	14 (3.7)
Lazio	1.11*^⋆^	0.55*	0.25*	1.29*	13 (3.6)	18 (4.2)
Liguria	1.10*	0.92*	-	1.50*	12 (3.5)	18 (4.2)
Lombardia	1.03*	0.55*	-	1.34*	27 (5.2)	31 (5.6)
Toscana	-	-0.84*	-	2.44*	25 (5.1)	29 (5.4)

We have also back-tested this model, and the quality of results is comparable to what we have seen for non-birth-related events.

### Amounts

A large quantity of results is obtained when looking at the amounts associated with all types of claims. Here we show how the model-produced information should be interpreted and used, by only focusing on the results obtained for the amounts associated with injuries, in our opinion the most interesting ones.

Here, the departments OT and NA are not pooled together, because it may be relevant to isolate the amounts related to non-major departments (OT), from those for which no information was available (NA).

#### Additional descriptive statistics and model forecasts

The cost analyses for injuries are based on a large number of claims (11134), shown by region and department in Table 12. A total of 38.1% of such claims had an associated amount equal to zero.

**Table 12:** Number of claims by department (columns) and geographic region (rows), as used in the analysis of the monetary amounts related to injuries’ claims.

Department	OT	AN	SU	ME	NA	OR	GY	NC	HS	ED	Tot.
Calabria	1	0	9	0	39	0	1	6	1	3	*60*
Campania	0	0	0	0	0	0	0	1	0	0	*1*
Emilia-Romagna	5	29	183	58	0	141	30	88	39	128	*701*
Lazio	8	4	58	22	38	27	14	79	20	43	*313*
Liguria	240	98	574	173	0	427	88	144	107	277	*2128*
Lombardia	64	164	1286	513	1	741	340	516	165	736	*4526*
Marche	0	4	138	4	173	4	6	17	0	4	*350*
Nordest	455	9	262	109	2	156	33	69	32	133	*1260*
Puglia	1	0	0	0	3	0	0	0	0	0	*4*
Sicilia	16	8	48	14	0	24	20	59	6	13	*208*
Toscana	21	37	129	54	0	83	23	113	75	64	*599*
Umbria	19	3	78	16	331	13	11	28	5	16	*520*
Valle D’Aosta	4	10	23	8	0	29	9	8	12	17	*120*
Veneto	17	22	101	31	0	41	20	39	32	41	*344*
Tot.	*851*	*388*	*2889*	*1002*	*587*	*1686*	*595*	*1167*	*494*	*1475*	*11134*

For injuries, the model selection procedure for the probability that cost is equal to zero has identified statistically significant effects for several regions, medical departments, and for calendar time (quadratic effect). For the conditional (on its being positive) model for cost, the model selection process identified significant effects for the Sicilia and Veneto regions. Detailed results, including all parameter estimates, are reported in Appendix 3. Note that from a health management point of view it would be interesting to further investigate these regional differences. Despite being both part of the Italian Health System, Veneto and Sicilia have two very different sanitary management systems, in accordance with the Italian law, which provides regions with a high level of independence.

Tables 13 and 14 contain descriptive statistics for the injury claims, for each of the regions and departments as identified by the models. In particular the tables show: the total number of claims used for the analysis (*n*); for positive amounts, their observed conditional mean and median (*C-Mean* and *C-Median*) and the conditional mean and variance of their natural logarithm (*C-LogMean* and *C-LogVar*); and the overall (i.e. unconditional) observed mean and median (*Mean* and *Median*).

**Table 13:** Descriptive statistics of amounts by region identified as significant by the model.

Region	n	C-Mean	C-LogMean	C-LogVar	C-Median	Mean	Median
Other	1286	36323.45	9.317607	2.761094	12652.756	14037.91	0
Lazio	313	47798.19	9.266628	2.674436	10622.88	25197.13	881.0123
Liguria	2128	27104.58	9.098354	2.359347	9058.494	17513.53	3230.3015
Lombardia	4526	46277.29	9.211569	2.968043	10558.519	33936	4920.1239
Marche	350	36803.6	9.385524	1.837179	10543.726	19137.87	1288.7663
Nordest	1260	30683.63	8.893184	3.26288	8293.666	14976.53	0
Sicilia	208	20616.81	8.470482	2.184822	4970.146	12786.39	1432.8085
Toscana	599	32205.8	8.835486	2.644944	6482.19	16828.74	389.9262
Valle D’Aosta	120	32956.62	8.924531	3.755072	9194.341	16752.95	122.2404
Veneto	344	24764.69	8.442321	3.622398	5547.704	16989.73	1096.6957
Total	11134	38155.26	9.114297	2.858998	9613.77	23618.29	2321.331

**Table 14:** Descriptive statistics of amounts by department identified as significant by the model.

Department	n	C-Mean	C-LogMean	C-LogVar	C-Median	Mean	Median
OT	851	22837.68	8.60146	3.149753	5178.526	10251.461	0
AN	388	14849.7	7.897819	2.568422	2769.462	11022.456	1368.6551
SU	2889	51714.51	9.43135	3.09725	13588.347	34905.95	5095.9336
OR	1686	40519.36	9.668339	1.972114	16498.79	29872.816	8808.9418
GY	595	62471.77	9.662451	2.21825	14388.323	43257.763	7766.3733
HS	494	28074.94	8.653037	3.233145	5821.155	16310.745	668.0726
ED	1475	24507.16	8.828775	2.155698	7163.041	15584.892	2411.4681
ME	1002	52625.46	9.010246	3.261793	7403.821	29411.437	875.6562
NA	587	34289.65	9.268644	2.348948	10197.789	11274.108	0
NC	1167	9209.32	8.241314	2.013242	4356.658	5042.635	515.4576
Total	11134	38155.26	9.114297	2.858998	9613.77	23618.29	2321.331

Thanks to our modeling, one may compute estimates for all relevant model-based quantities for any specific time point, as long as it is not too far from the time window of data collection. Table 15 provides a detailed legend of the information that is presented in Tables 16 and 17, where forecasts for June 30 2013 are provided (remember that our data stop on December 31st 2012, therefore June 30 2013 is a future date).

**Table 15:** Legend of quantities from the models for the monetary amounts (“costs” for insurance companies).

Label	Meaning
P(C>0)	Estimated *P*(*C* > 0)
ECost	Estimated mean cost (with 95% Confidence Interval—CI)
Median	Estimated median cost (with 95% CI)
*q*_0.90_	Estimated 90th quantile of cost (with 95% CI), also known as Value-at-Risk in risk management, or *V aR*_0.90_
*ES*_0.90_	Estimated Expected Shortfall above the 90th quantile of cost

**Table 16:** Forecast probability of non-zero amounts, median amounts (with 95% confidence interval), expected amounts (with 95% confidence interval), 90th quantiles (with 95% confidence interval), and conditional 10%-tail expected amount (shortfall), by region x department as identified by the models. Forecasts refer to 30 June 2013.

Region	Dept	P(C>0)	Median	CI Median	ECost	CI ECost	*q*_0.90_	CI *q*_0.90_	*ES*_0.90_
Other	OT	0.65	793.0	(239.2, 1699.9)	6134.4	(3752.1, 9702.2)	13419.1	(8122.6, 21273.9)	44186.5
Other	AN	0.83	826.1	(430, 1426.9)	3762.3	(2319.6, 5913.4)	8229.7	(5085.6, 12894.1)	25074.8
Other	SU	0.77	3206.9	(1682.5, 5441)	16304.0	(10595.4, 24421.4)	35730.3	(23224.9, 53399.1)	111048.0
Other	OR	0.83	4732.6	(2699.5, 7660.9)	21589.7	(14182.8, 32094.8)	47227.3	(31090.4, 70014.2)	143941.1
Other	GY	0.77	3986.4	(1915.4, 7154.2)	20438.0	(12646.5, 31949)	44793.7	(27711.5, 69849)	139414.9
Other	HS	0.71	1101.5	(432.6, 2140)	6709.1	(4119.8, 10507)	14708.8	(8985.1, 23016.5)	47010.0
Other	ED	0.74	1499.2	(697.8, 2715.4)	8372.0	(5287.4, 12847.2)	18357.0	(11569.2, 28125.5)	57925.3
Other	ME	0.65	1151.2	(343.2, 2496.7)	8904.9	(5383.3, 14249.7)	19479.5	(11653.8, 31245.2)	64142.4
Other	NA	0.65	1492.4	(420.9, 3421.5)	11544.6	(6602.1, 19528.3)	25253.8	(14292.5, 42819.5)	83156.0
Other	NC	0.65	555.8	(166.8, 1197.5)	4299.6	(2616.6, 6834.5)	9405.3	(5664.5, 14985.9)	30970.0
Lazio	OT	0.79	1549.4	(786, 2659.9)	7529.6	(4781.4, 11448.6)	16491.8	(10482.6, 25000.6)	50854.8
Lazio	AN	0.91	1035.2	(609, 1655.1)	4139.4	(2644.7, 6323.6)	9010.6	(5777, 13728.2)	26725.2
Lazio	SU	0.88	4423.6	(2677.9, 6830.6)	18507.6	(12417.2, 26925.4)	40371.7	(27172.9, 58564.8)	120925.2
Lazio	OR	0.91	5939.5	(3732.7, 8991.6)	23766.2	(16057.8, 34487.4)	51735.4	(35065.2, 74889.4)	153470.1
Lazio	GY	0.87	5538.5	(3174.5, 8925.5)	23250.2	(14955.7, 35140.3)	50724.0	(32738.8, 76427.6)	152041.2
Lazio	HS	0.83	1768.4	(955.3, 2915.6)	7926.8	(5092.7, 11909.4)	17331.8	(11164.8, 25951.4)	52645.4
Lazio	ED	0.85	2239.1	(1283, 3580.8)	9717.6	(6367.3, 14409.4)	21226.6	(13950.2, 31376.2)	64063.8
Lazio	ME	0.79	2249.1	(1127.7, 3906.6)	10930.2	(6860.1, 16814.7)	23939.9	(15039.8, 36718.6)	73822.4
Lazio	NA	0.79	2915.8	(1383, 5353.7)	14170.3	(8413.3, 23043.4)	31036.4	(18445.1, 50320.3)	95705.3
Lazio	NC	0.79	1086.0	(548.1, 1873.7)	5277.5	(3334.4, 8064.7)	11559.0	(7310.3, 17611)	35643.8
Liguria	OT	0.85	1840.6	(1088.9, 2916.8)	8057.2	(5336.5, 11911.5)	17604.5	(11689.8, 25955.6)	53224.0
Liguria	AN	0.94	1103.7	(689.2, 1710)	4262.4	(2789.3, 6422.1)	9259.9	(6075.3, 13925.8)	27247.8
Liguria	SU	0.91	4840.0	(3160.4, 7166.9)	19256.8	(13288.9, 27529.3)	41906.5	(28993.8, 59785.4)	124155.5
Liguria	OR	0.94	6334.9	(4205.2, 9303.6)	24476.6	(16908.9, 35047.5)	53175.5	(36817.2, 76013.3)	156489.5
Liguria	GY	0.91	6071.4	(3775.1, 9366.9)	24209.1	(16041.7, 35933)	52689.8	(35014.3, 78029.3)	156179.8
Liguria	HS	0.88	2010.6	(1222.2, 3118)	8363.7	(5578.4, 12273.3)	18240.0	(12202.9, 26693.2)	54567.4
Liguria	ED	0.90	2500.7	(1576.1, 3797.9)	10189.1	(6898.7, 14799.6)	22200.0	(15073.2, 32168.9)	66118.7
Liguria	ME	0.85	2671.8	(1562.3, 4283.9)	11696.0	(7656.6, 17494.5)	25555.2	(16771.9, 38121.2)	77261.6
Liguria	NA	0.85	3463.8	(1916.1, 5870.8)	15163.0	(9390.2, 23975)	33130.5	(20569.4, 52242.5)	100164.0
Liguria	NC	0.85	1290.0	(759.4, 2054.7)	5647.2	(3721.6, 8390.8)	12338.9	(8152.2, 18283.8)	37304.4
Lombardia	OT	0.89	2060.5	(1301.9, 3134.9)	8453.6	(5722.5, 12303.7)	18424.6	(12505, 26754.8)	54956.6
Lombardia	AN	0.95	1151.9	(740.1, 1754.5)	4348.9	(2880.7, 6502)	9433.7	(6261.2, 14085)	27611.1
Lombardia	SU	0.94	5138.5	(3471.1, 7446.6)	19793.2	(13848.1, 28031.3)	42992.5	(30140.5, 60793)	126431.2
Lombardia	OR	0.95	6613.3	(4499.7, 9561.5)	24976.3	(17438, 35510.3)	54179.8	(37891.1, 76937.2)	158588.4
Lombardia	GY	0.94	6453.9	(4172.6, 9724)	24896.4	(16757.3, 36573.8)	54082.2	(36484.8, 79315)	159098.3
Lombardia	HS	0.91	2188.7	(1404.3, 3285.4)	8684.2	(5907.2, 12574)	18895.6	(12888.6, 27300.5)	55946.8
Lombardia	ED	0.93	2690.9	(1771.1, 3977.7)	10531.0	(7250.2, 15122.5)	22896.4	(15800.2, 32819.3)	67581.0
Lombardia	ME	0.89	2991.1	(1867.9, 4604.3)	12271.5	(8210.3, 18070.5)	26745.7	(17941.5, 39295.1)	79776.6
Lombardia	NA	0.89	3877.7	(2290.8, 6309.8)	15909.0	(10069.3, 24764.4)	34673.9	(22003.8, 53851.2)	103424.6
Lombardia	NC	0.89	1444.2	(907.9, 2208.3)	5925.1	(3990.7, 8667)	12913.7	(8720.7, 18846.8)	38518.7
Marche	OT	0.80	1561.8	(799.6, 2669.9)	7552.3	(4806.4, 11466.6)	16540.0	(10538, 25038)	50958.0
Marche	AN	0.91	1038.3	(612.4, 1657.6)	4144.9	(2650.8, 6328.2)	9021.7	(5789.8, 13737.5)	26748.6
Marche	SU	0.88	4441.9	(2703.3, 6841.2)	18540.6	(12463.2, 26944.4)	40439.8	(27270.1, 58603.4)	121068.7
Marche	OR	0.91	5957.1	(3750.2, 9008.5)	23797.8	(16089.5, 34517.7)	51799.7	(35130.9, 74950.3)	153605.3
Marche	GY	0.88	5561.9	(3196.7, 8948.8)	23292.4	(14996, 35182)	50811.1	(32824.3, 76512.2)	152225.0
Marche	HS	0.84	1778.9	(962.6, 2928.6)	7945.8	(5106, 11932.7)	17371.6	(11193.7, 25999.2)	52730.0
Marche	ED	0.86	2250.5	(1292.3, 3593.7)	9738.3	(6384.2, 14432.6)	21269.5	(13986.3, 31423.5)	64154.7
Marche	ME	0.80	2267.2	(1147.2, 3921.2)	10963.1	(6896, 16841.2)	24010.0	(15119.4, 36773.6)	73972.1
Marche	NA	0.80	2939.3	(1406.9, 5373.8)	14212.8	(8457.4, 23079.7)	31127.2	(18542.7, 50395.6)	95899.4
Marche	NC	0.80	1094.7	(557.6, 1880.7)	5293.3	(3351.9, 8077.4)	11592.8	(7349, 17637.4)	35716.1

**Table 17:** Forecast probability of non-zero amounts, median amounts (with 95% confidence interval), expected amounts (with 95% confidence interval), 90th quantiles (with 95% confidence interval), and conditional 10%-tail expected amount (shortfall), by region x department as identified by the models. Forecasts refer to 30 June 2013. (Continued).

Region	Dept	P(C>0)	Median	CI Median	ECost	CI ECost	*q*_0.90_	CI *q*_0.90_	*ES*_0.90_
Nordest	OT	0.76	1397.2	(704.7, 2432.8)	7252.4	(4630.8, 11038.3)	15896.7	(10147.6, 24143)	49578.5
Nordest	AN	0.89	997.1	(583, 1607.1)	4070.9	(2597.7, 6237.4)	8870.4	(5679, 13554.6)	26430.5
Nordest	SU	0.86	4195.6	(2539.9, 6521.4)	18096.5	(12166.9, 26369.5)	39520.6	(26642.2, 57432.8)	119126.4
Nordest	OR	0.89	5719.4	(3593.2, 8702.5)	23370.2	(15806, 33968.2)	50926.0	(34540.7, 73841.7)	151768.1
Nordest	GY	0.85	5247.1	(2989.2, 8540.1)	22724.7	(14619.1, 34447.4)	49634.9	(32022.5, 75017.2)	149738.6
Nordest	HS	0.81	1638.9	(873.8, 2743.7)	7692.1	(4943.2, 11599.8)	16837.2	(10838.8, 25313.9)	51592.7
Nordest	ED	0.83	2097.6	(1197.9, 3387.1)	9462.0	(6212.1, 14061)	20692.2	(13616.3, 30662)	62930.0
Nordest	ME	0.76	2028.3	(1011.1, 3573.1)	10527.8	(6644, 16212.1)	23076.1	(14559.2, 35459.1)	71969.6
Nordest	NA	0.76	2629.5	(1240.1, 4896.6)	13648.5	(8148.4, 22217.6)	29916.5	(17855.7, 48594.2)	93303.4
Nordest	NC	0.76	979.3	(491.5, 1713.7)	5083.2	(3229.4, 7775.7)	11141.9	(7076.7, 17007)	34749.3
Sicilia	OT	0.85	1024.1	(498.5, 1899.6)	4441.6	(2507.7, 7606.8)	9701.7	(5495, 16559.5)	29276.5
Sicilia	AN	0.94	608.1	(328.8, 1073)	2340.8	(1340.8, 4005.5)	5084.3	(2921.3, 8681.8)	14949.7
Sicilia	SU	0.92	2672.9	(1469.7, 4608.4)	10586.1	(6258, 17525.1)	23031.5	(13660.4, 38034.4)	68164.6
Sicilia	OR	0.94	3490.2	(1972.4, 5935.6)	13442.1	(8000, 22204.2)	29197.3	(17426.7, 48134)	85859.7
Sicilia	GY	0.91	3353.5	(1781.7, 5941.5)	13309.4	(7654.7, 22586.1)	28960.0	(16714.7, 49016.8)	85750.9
Sicilia	HS	0.89	1114.3	(566.2, 2009)	4604.1	(2630.7, 7803.4)	10037.9	(5757.1, 16958.5)	29986.8
Sicilia	ED	0.90	1383.6	(729.6, 2450.4)	5605.5	(3244.8, 9432.6)	12209.9	(7093, 20487.6)	36319.3
Sicilia	ME	0.85	1486.6	(717.1, 2782.6)	6447.6	(3607.4, 11142.6)	14083.3	(7904.9, 24256.7)	42498.7
Sicilia	NA	0.85	1927.3	(889.7, 3769.7)	8358.8	(4475.5, 15095.4)	18258.0	(9806.9, 32861.7)	55096.5
Sicilia	NC	0.85	717.8	(350.9, 1325.6)	3113.1	(1765.3, 5308.4)	6799.9	(3868.2, 11556.1)	20519.7
Toscana	OT	0.79	1525.6	(777, 2620.5)	7486.4	(4764.8, 11377.7)	16399.5	(10445.9, 24853)	50657.3
Toscana	AN	0.91	1029.4	(607.7, 1645.3)	4128.9	(2642.4, 6306)	8989.2	(5772.3, 13692.9)	26680.2
Toscana	SU	0.87	4388.5	(2666.2, 6774.4)	18444.3	(12396, 26824.3)	40241.1	(27128.1, 58359.6)	120649.5
Toscana	OR	0.91	5905.8	(3721.6, 8938.5)	23705.6	(16037.8, 34392.1)	51611.7	(35023.6, 74697.5)	153210.2
Toscana	GY	0.87	5493.6	(3156, 8856.8)	23169.3	(14922.2, 35016.8)	50556.9	(32667.8, 76177)	151688.2
Toscana	HS	0.83	1748.3	(951.7, 2879.8)	7890.4	(5086.1, 11845)	17255.5	(11150.4, 25819.1)	52483.3
Toscana	ED	0.85	2217.2	(1274.4, 3546.6)	9678.2	(6351.7, 14347.9)	21144.4	(13916.7, 31250.5)	63889.6
Toscana	ME	0.79	2214.6	(1114.8, 3848.8)	10867.5	(6836.3, 16710.5)	23805.9	(14987.2, 36501.8)	73535.6
Toscana	NA	0.79	2871.1	(1367.3, 5274.5)	14088.9	(8384.2, 22900.6)	30862.7	(18380.6, 50023.3)	95333.5
Toscana	NC	0.79	1069.3	(541.9, 1846)	5247.2	(3322.9, 8014.7)	11494.3	(7284.7, 17507.1)	35505.4
Valle D’Aosta	OT	0.75	1311.1	(486.4, 2522.8)	7094.8	(4221.6, 11201.1)	15555.1	(9220.1, 24484.4)	48842.7
Valle D’Aosta	AN	0.88	974.7	(524.6, 1619.4)	4030.6	(2492, 6259.5)	8787.7	(5456.1, 13599.1)	26256.3
Valle D’Aosta	SU	0.85	4063.0	(2163.2, 6636.8)	17857.2	(11480.2, 26577)	39022.0	(25167.5, 57856.3)	118070.2
Valle D’Aosta	OR	0.88	5590.1	(3219.6, 8801.8)	23137.6	(15129.9, 34146.6)	50448.4	(33118.5, 74202.3)	150762.0
Valle D’Aosta	GY	0.84	5077.7	(2545.2, 8661.5)	22418.9	(13808.7, 34665.7)	48997.3	(30275.4, 75462.6)	148387.3
Valle D’Aosta	HS	0.80	1564.5	(688.9, 2795.9)	7557.1	(4601.3, 11693.9)	16550.4	(10081.6, 25508.4)	50980.3
Valle D’Aosta	ED	0.82	2016.0	(978.7, 3456.4)	9314.2	(5809.9, 14185.7)	20380.9	(12738.5, 30918.3)	62267.6
Valle D’Aosta	ME	0.75	1903.2	(697.9, 3705.2)	10299.0	(6057, 16451.1)	22580.2	(13228.5, 35960.5)	70901.6
Valle D’Aosta	NA	0.75	2467.4	(855.9, 5077.8)	13351.9	(7428.4, 22545.1)	29273.6	(16223.7, 49281.4)	91918.7
Valle D’Aosta	NC	0.75	918.9	(339.2, 1777.1)	4972.7	(2944.1, 7890.3)	10902.4	(6429.9, 17247.5)	34233.6
Veneto	OT	0.88	1005.8	(554.3, 1703.8)	4204.4	(2570.5, 6709.3)	9171.0	(5625.1, 14592.5)	27465.0
Veneto	AN	0.95	573.9	(335.8, 949.2)	2181.3	(1331, 3523.3)	4733.8	(2895.8, 7633.3)	13877.2
Veneto	SU	0.93	2547.4	(1534.6, 4078)	9905.7	(6283.8, 15378.2)	21528.6	(13694.3, 33355.7)	63450.1
Veneto	OR	0.95	3294.8	(2015.9, 5239.5)	12526.9	(7957, 19483.8)	27186.4	(17308.3, 42218.1)	79703.7
Veneto	GY	0.93	3198.4	(1854.3, 5287.3)	12457.7	(7650.9, 19926.3)	27077.8	(16679.5, 43218.7)	79835.8
Veneto	HS	0.90	1077.1	(612.5, 1785.6)	4332.8	(2675.6, 6856.3)	9434.4	(5845.8, 14889.5)	28019.7
Veneto	ED	0.92	1328.8	(774.9, 2175.2)	5261.6	(3278.1, 8287.8)	11447.2	(7154, 17989.1)	33878.0
Veneto	ME	0.88	1460.0	(795, 2503.5)	6103.2	(3686.4, 9858.4)	13312.9	(8067, 21441.7)	39868.9
Veneto	NA	0.88	1892.8	(980.5, 3411.5)	7912.4	(4546.7, 13434.1)	17259.2	(9949.6, 29218.6)	51687.2
Veneto	NC	0.88	704.9	(386.3, 1201.1)	2946.8	(1791.2, 4729.9)	6427.9	(3919.8, 10287.2)	19250.0

Let us focus our attention on Table 16 and, in particular, on the Liguria region. The departments of Anesthesia and Orthopedics are the ones with the highest probability of non-zero amounts, that is to say those departments that generate the largest number of positive disbursements for insurance companies and hospitals. The departments showing the highest median amounts are Orthopedics and Obstetrics. These departments are also the ones associated with the highest expected costs (about 24k euros), the highest 90% Value-at-Risk (the amount with respect to which only 10% of all paid amounts are larger, i.e. the 90% quantile) and, as a consequence, the highest 90% expected shortfall, that is to say the expected paid amount, when considering the top 10% of all disbursements.

Similar considerations can be made for all the regions in the data set, and it is interesting to see how, in every region, Orthopedics and Obstetrics appear to be the most expensive departments in terms of disbursement, every time a medical malpractice claim is made. The NC category (the whole hospital), on the contrary, is on average associated to the smallest amounts. This is easy to explain: the NC category typically refers to events happening in the common areas of the hospital, which are usually associated to monetary damages and minor injuries.

It is also possible to plot the model-based quantities of interest with respect to time, in order to study their trends for different covariate values. Such plots are useful to obtain an exploratory overall impression of the absolute impact of the baseline covariates and time on the cost associated with the claims.

**Fig 3:**
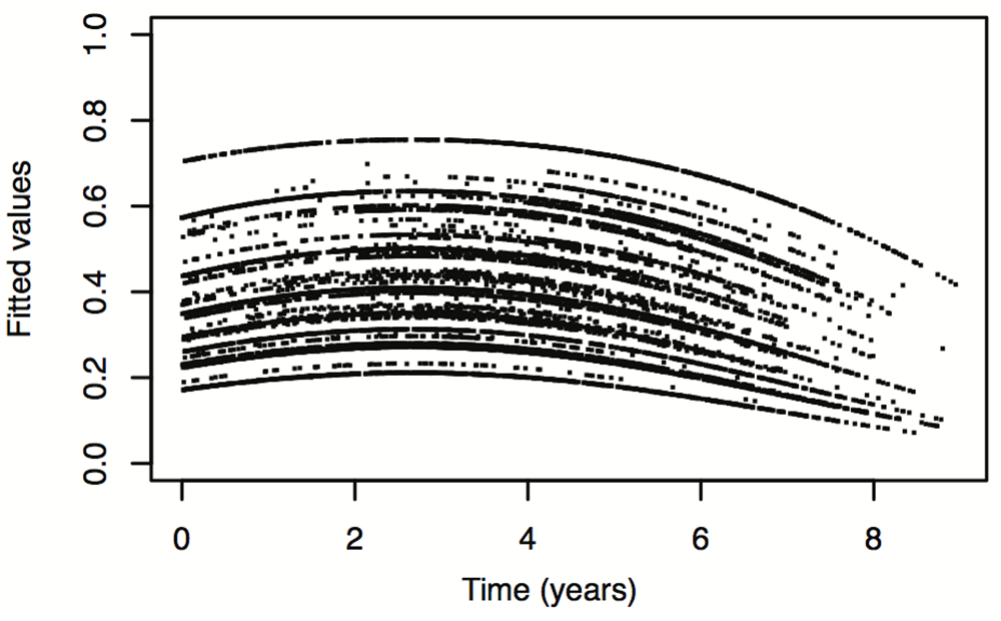
Estimated probability that Cost is equal to zero vs. time. Estimated probability that Cost is equal to zero vs. time, for all baseline covariate values.

**Fig 4:**
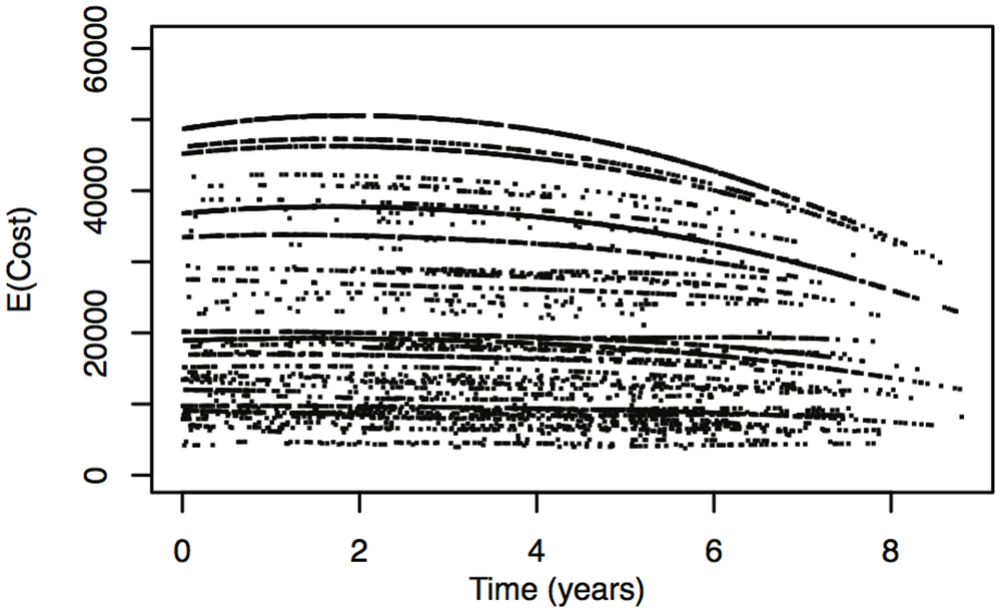
Estimated unconditional mean Cost vs. time. Estimated unconditional mean Cost vs. time, for all baseline covariate values.

**Fig 5:**
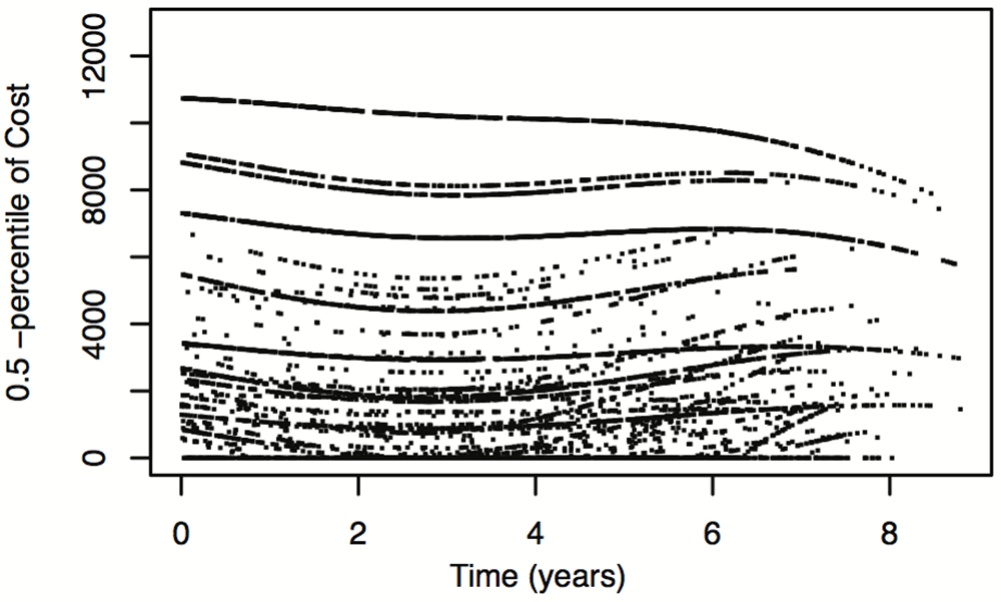
Estimated unconditional median Cost vs. time. Estimated unconditional median Cost vs. time, for all baseline covariate values.

While the object of such detailed examinations is not among the goals of this article, we do show in Figs 3–5 three examples of such model-based curves. Fig 3 shows the estimated probability that cost is equal to zero versus time from January 1st, 2004. Figs 4 and 5 show, again against time, the estimated median cost and the 95*th* quantile of the cost distribution, also taking into consideration the zero amounts. The different curves on the three plots correspond to the different combinations of baseline covariate values (regions by departments). In Fig 3 a consistent behavior is identifiable for all regions by departments: the estimated probability that cost is equal to zero tends to slightly increase during the first 30 months and then decreases. For Figs 4 and 5, on the contrary, no unique trend is observable and further analyses are needed.

#### Expected and tail amounts

We finally provide some examples to show how to derive expected and tail amounts.

For 2013, a total of 218 injury-type claims have been forecast for the orthopedic departments of the Lombardia region. The corresponding average cost of each of such events is equal to 24,976 euros. A simple multiplication of such average amount by 218 generates an estimated overall cost for such claims of 5,444,768 euros. It should be noted that the 95% confidence interval for the claim-specific expected cost, i.e. (17,438;35,510), is all but narrow, and that the overall cost forecast also has its own sampling variability. From the part of the model that describes the probability that the amounts are equal to zero, one may easily produce a forecast for the proportion of claims (out of the 218) that will have a strictly positive amount. For 2013 such proportion is equal to 0.95, and the 95% confidence interval is (0.94, 0.97). As a consequence, a total of 207 injury-type claims with non-zero associated amounts are expected, and the 95% confidence interval is derived as (205, 211).

Focusing on the extreme amounts and on the number of such claims, let us now consider the case of injuries in anesthesia departments of the Toscana region. A total of 34 claims have been forecast for 2013, and the June 30 2013 forecast for the 90*th* quantile of the amount distribution is equal to 8,989. This forecast already takes into account the zero amounts, which are estimated to occur with probability equal to 1 − 0.91 = 0.09. The binomial formula in Appendix 2.3 allows us to easily obtain the probability that at least 8 of the 34 claims have associated amounts greater than or equal to 8,989 as being less than or equal to 0.017. Note that in this example *np*(1 − *p*) = 3.06, so that it would not be appropriate to use the normal approximation for the previous computations. A similar procedure can easily be employed for the number of claims that may yield even more extreme amounts; it is in fact sufficient to use larger quantiles of the amount distribution.

Finally, for the same departments and for the same year, the expected amount for claims that have an amount greater than the 90*th* quantile (8,989 euros) is estimated as being equal to 26,681. Such number is quite large since it refers to amounts that are in the top 10% tail of the distribution. As we have pointed out above, such an amount should be treated with caution as it is based on our parametric (lognormal and logistic) assumptions.

## Discussion

The problem of medical malpractice risk assessment is becoming more and more important for the Italian Health System, because of its implications in terms of public expenditure and hospital management. Indeed, differently from the past, an increasing number of Italian patients is following the North American trend of filing lawsuits against hospitals and doctors [8]. Relatedly, there has recently been a lot of discussion in the country about an advertising campaign on TV and newspapers. The campaign suggested the possibility for patients to be reimbursed for cases of medical malpractice. Notably, the campaign was promoted by some associations of lawyers, and it has caused a strong negative reaction from physicians in the country [19, 20].

In this article we have analyzed the number and the payoff amounts of medical malpractice claims in Italy, in the period 2004–2012, using a large database pooling the observations of three major international brokers. We believe this work will provide a useful contribution to the quantitative study of the phenomenon of medical malpractice, not only in Italy, but also in other countries.

Despite the richness of our data set, we stress once again that it is not advisable to extend any forecast based on our data to the whole country. As already observed, our data were not randomly sampled, as our observations only come from those hospitals, which have underwritten an insurance contract with one of the three brokers providing the database. This necessarily determines a selection bias, which undermines representativeness.

Our analysis seems to suggest an increase in the number of reported claims over time for most Italian regions (only exception: Lombardia), even if it will be interesting to observe whether this trend will continue in the future. The performances of the inhomogeneous Poisson process have been checked in-sample and via back-testing, and they have proved to be very satisfactory.

For what concerns the payoff amounts (for the settled claims), we have registered an average increase for claims due to death, a stationary behavior for claims due to injuries at birth and monetary damages, and a slight decrease for injuries.

We should point out that the expected values estimated for the costs in the different subcategories prove to be somewhat unstable, with wide prediction intervals. Nevertheless, these forecasts do provide useful indications, e.g. for the trend of costs over time. Clearly, the forecasts of the cost distribution’s quantiles are sensitive to the parametric model chosen (log-normal), as are the expected values predicted for the tails of the distributions for the various amounts. These are in fact dependent on the hypotheses made for the tails of the distributions. Once again, extreme caution should be used when interpreting such cost quantiles.

However, despite all caveats, we do think that our modeling has achieved its goal, in describing and forecasting the phenomenon of medical malpractice in Italy. Should a complete, more updated data set be made available, the methodology could be effectively employed to produce estimates for future periods of time.

Given our results about costs, not as high as one could expect, the decision taken by some Italian regions to consider the partial retention of the clinical risk is understandable. Naturally, this decision implies the necessity of acquiring properly skilled personnel, with the competence to deal with the process of accepting, assessing and—should this be required—settling claims for damage. They should also be qualified for defining the right policies for earmarking reserves in the public budget. Further, any decision to mitigate the clinical risk using insurance options should not be undertaken without first making a historical analysis of the claims experienced by each region, hospital and department. But, in order not to fall in the trick of historical bias, these decisions should also be oriented towards covering risks with the lowest frequency and the greatest financial impact—the so-called black swans in the everyday language. If said risks were not adequately covered by setting aside considerable budget reserves, the result would be a series of unforeseeable, and thus unmanageable, losses [14].

To conclude, some relevant points for discussion and future work include the possibility of implementing the methods on a continuous-time scale, so that a timely monitoring of the phenomenon could be performed. It could also be relevant to develop a related alarm system [21], as a way of monitoring the phenomenon [22].

As time progresses, further checks on the accuracy of the models’ forecasts may then be performed, by matching our prediction with the newly observed data made available by continuous monitoring.

Also, while such information was not available to us, a possible enrichment of the analyses could include the variation of the number of patients and of CMI over time, within each “department by hospital” event-generating unit.

Finally, the lack of regional homogeneity observed in this analysis could serve as a starting point for a more general discussion on the interpretation of these differences. If more data become available, it would be interesting to study the impact of the different regional Health Systems on medical malpractice claims in Italy.

## Appendix 1—On the lognormal assumption for the distribution of the amounts

In this appendix we report on some additional analyses that further support the use of the lognormal distribution in our analyses of the amounts.

**Fig 6:**
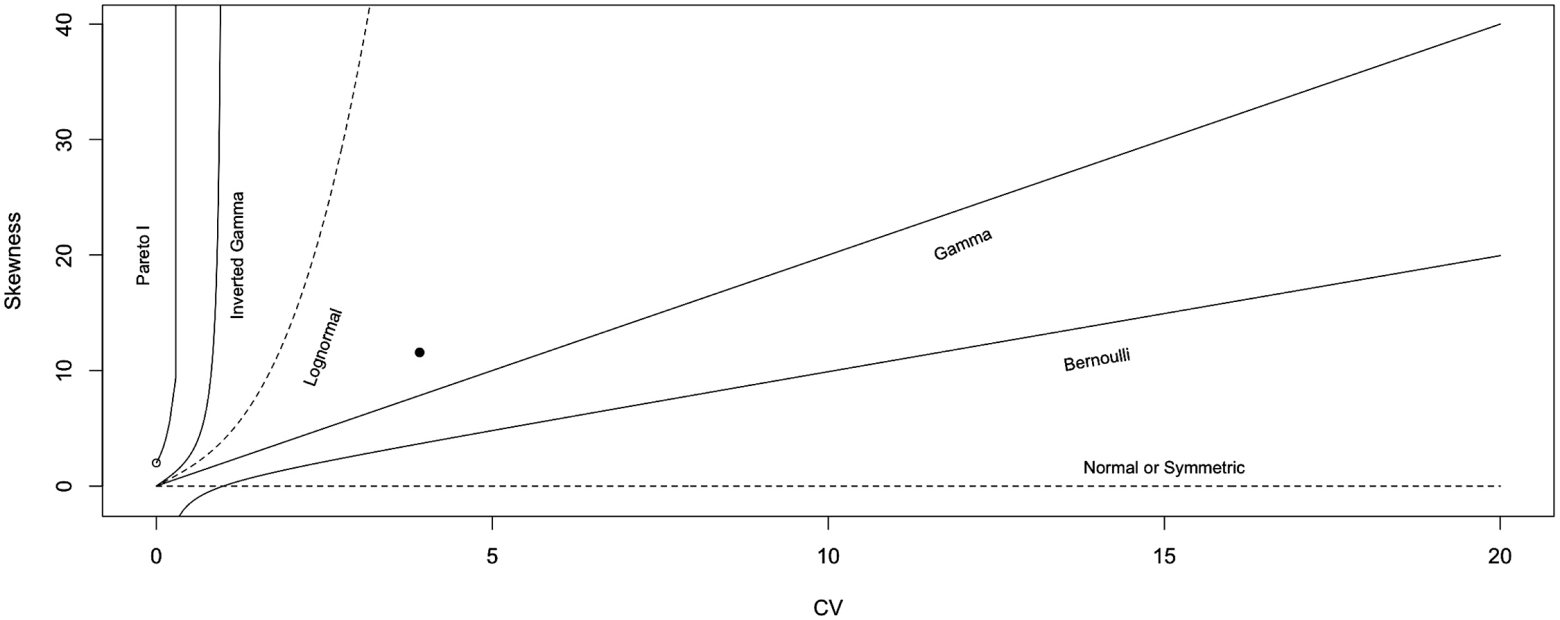
Discriminant Moment-ratio Plot. Discriminant moment-ratio plot for the non-zero payments, all claims pooled together. The large dot represents the pair “CV and Skewness” and it falls in the so-called lognormal region.

A moment-ratio plot, as the one in Fig 6, involving the sample coefficient of variation (CV) and the skewness, indicates that claims (pooled all together) can be modeled with a lognormal-like distribution. Introduced by [23], and further developed in [24] and [25], moment-ratio plots represent a simple way of visualizing and discriminating among distributions. Some distributions may be represented as a set of points, some others as curves or areas. For more details on the interpretation of moment-ratio plots we also refer to [26].

Lognormality is also supported by the study of the mean excess function of claims, a tool commonly used in extreme value statistics. In particular, let *X* be a random variable with distribution *F* and right endpoint *x*_*F*_ (i.e. $x_{F}=\sup\left\{x\in R:F\left(x\right)<1\right\}$). The function

$$e\left(u\right)=E\left[X-u|X>u\right]=\frac{\int_{u}^{\infty }\left(t-u\right)\text{d}F\left(t\right)}{\int_{u}^{\infty }\text{d}F\left(t\right)},\;0<u<x_{F},$$ (1)

is called mean excess function of *X* (ME). The empirical ME of a sample *X*_1_, *X*_2_,…, *X*_*n*_ is easily computed as

$$e_{n}\left(u\right)=\frac{\sum_{i=1}^{n}\left(X_{i}-u\right)}{\sum_{i=1}^{n}1_{\left\{X_{i}>u\right\}}},$$ (2)

that is the sum of the exceedances over the threshold *u* divided by the number of such data points. Interestingly, the ME is a way of characterizing distributions within the class of continuous distributions [27]. For example, the Pareto distribution (and its generalizations) is the only distribution characterized by the so-called van der Wijk’s law [28], that’s to say by a mean excess function linearly increasing in the threshold *u*.

**Fig 7:**
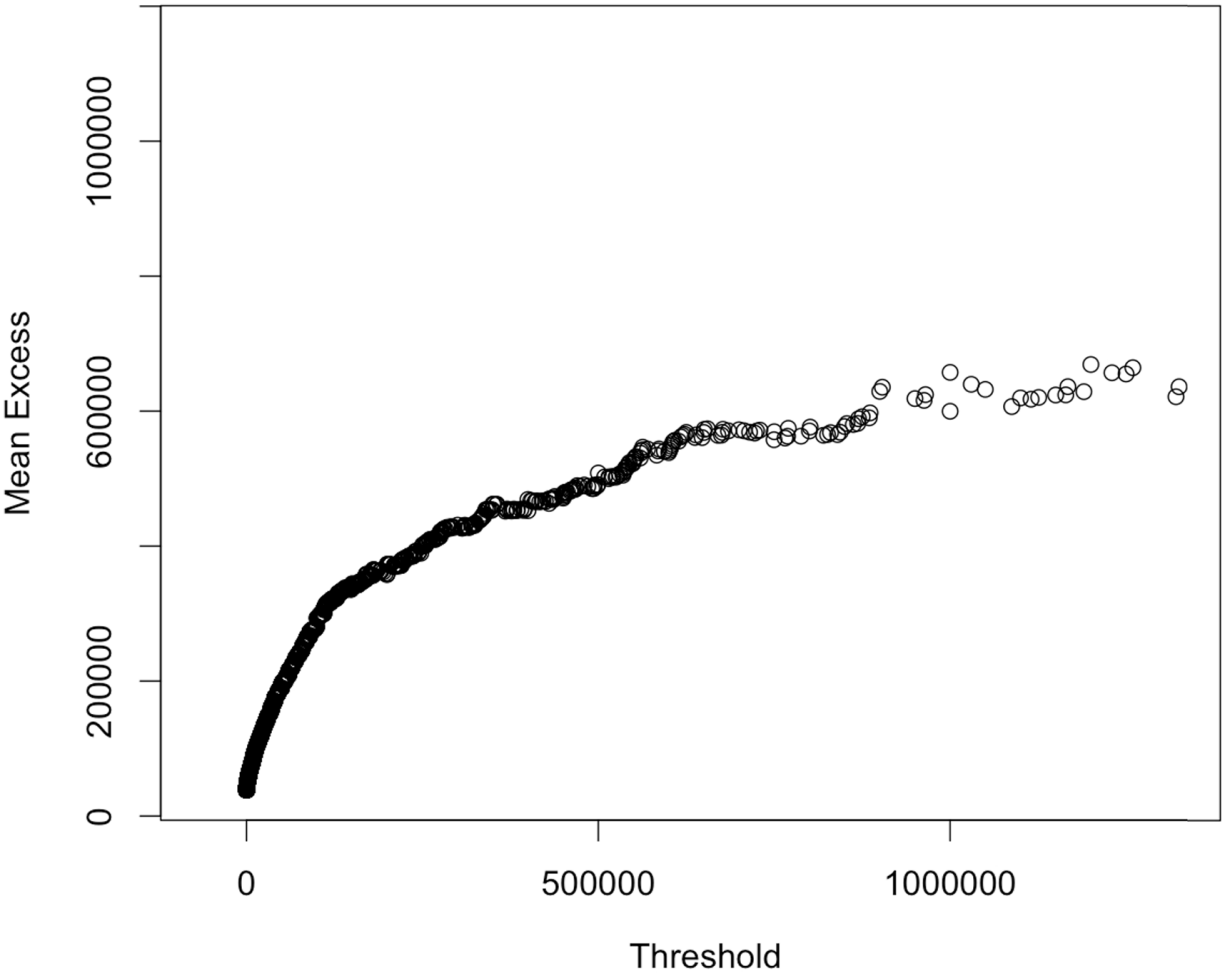
Mean Excess Function Plot. Mean excess function plot for the non-zero payments. Concavity is a symptom of lognormally distributed data.

In case of lognormally distributed random variables, we have

$$e_{LN}\left(u\right)=\frac{u\;\sigma^{2}}{\log(u)-\mu }\left(1+o\left(1\right)\right),$$ (3)

and the mean excess function has a behavior very similar to the sample plot computed on our data and shown in Fig 7. That graph is known as meplot, and it is obtained by plotting the pairs {(*X*_*i*: *n*_, *e*_*n*_(*X*_*i*: *n*_)) : *i* = 1, …, *n*}, where *X*_*i*: *n*_ is the *i*−th order statistic. For a complete treatment about mean excess functions and meplots we refer to [29].

**Fig 8:**
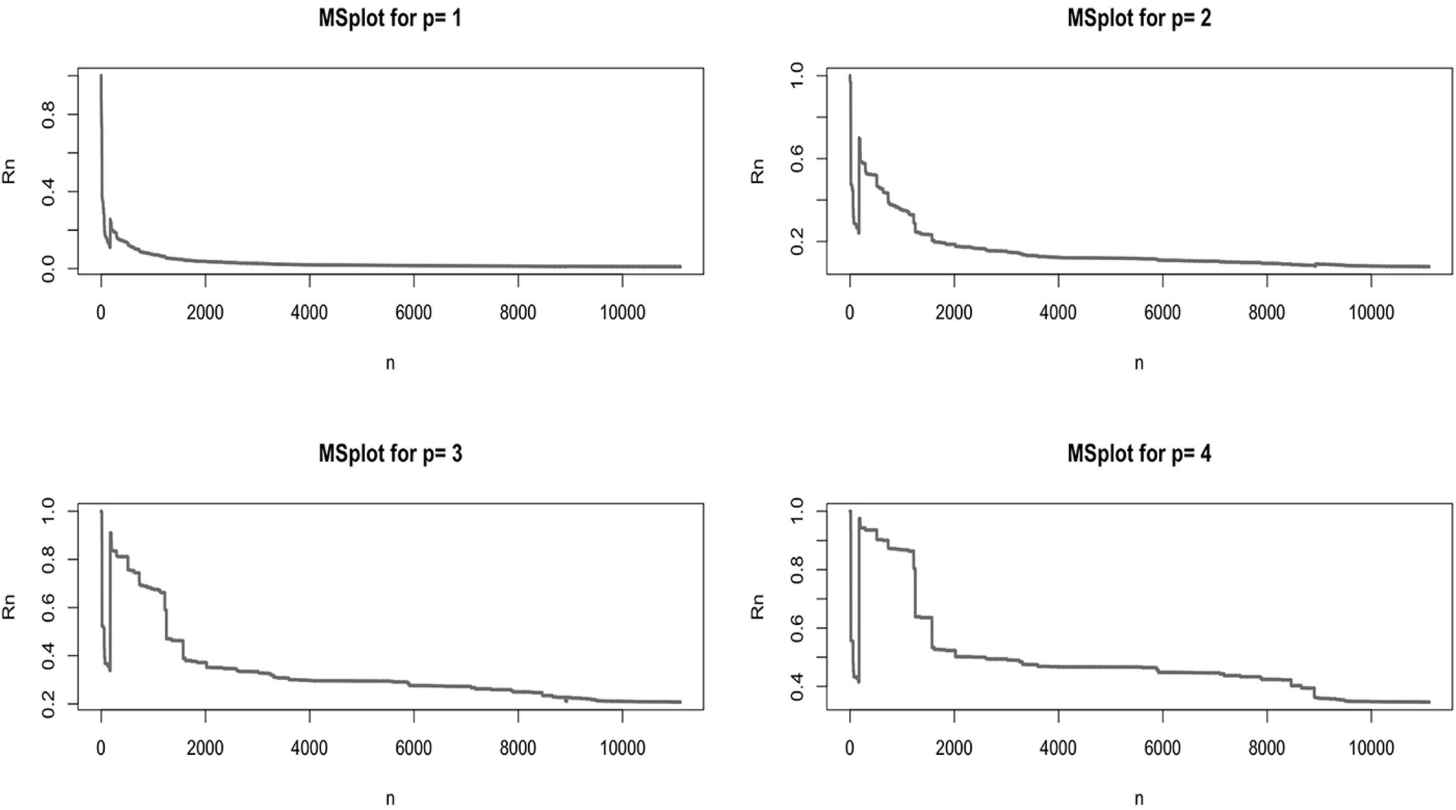
Maximum to Sum Plot. Maximum to Sum plot for the non-zero claims, first four moments (*p* = 1, …, 4). The convergence towards zero, in all four subplots, suggests that the corresponding moments are finite.

To further exclude other heavy-tailed models (such as the Generalized Pareto Regression [30]), we studied the finiteness of the first four moments for the non-zero payments. The use of a Maximum to Sum plot, as the one in Fig 8, shows that at least the first four moments of the distribution of claim amounts are finite, indicating the absence of very heavy tails. This plot relies on the fact that, for a sequence *X*_1_, *X*_2_, …, *X*_*n*_ of nonnegative i.i.d. random variables, if for *p* = 1, 2, 3…, *E*[*X*^*p*^] < ∞, then $R_{n}=M_{n}^{p}/S_{n}^{p}\to 0$ as *n* → ∞, where $S_{n}^{p}=\sum_{i=1}^{n}X_{i}^{p}$ and $M_{n}^{p}=\max\left(X_{1}^{p},...,X_{n}^{p}\right)$. This follows from the law of large numbers, as shown for example in [29]. In conclusion, in our case the existence of the first four moments suggests that Paretianity can safely be ruled out.

## Appendix 2—Technical details of models

### A2.1—On models for the number of events

For each unit of analysis *i*, *i* = 1, …, *m*, with *m* the number of units, we modeled the number of claims by an inhomogeneous Poisson process whose time-varying intensity function is linearly dependent on a set of covariates (including time itself).

For every *i* = 1, …, *m*, we let λ_*i*_(*t*) be the intensity of a Poisson process at time *t*, while Λ_*i*_(*t*) is the corresponding cumulative intensity, such that

$$\Lambda_{i}\left(t\right)=\int_{0}^{t}\lambda_{i}\left(u\right)\text{d}u.$$

We then assume the following functional form for the intensity function

$$\lambda_{i}\left(t\right)=\delta t^{\delta -1}\exp\left(x_{i}^{\prime }\gamma \right),$$ (4)

so that

$$\Lambda_{i}\left(t\right)=t^{\delta }\exp\left(x_{i}^{\prime }\gamma \right)$$ (5)

with

$$x_{i}^{\prime }\gamma =\gamma_{0}x_{i,0}+\gamma_{1}x_{i,1}+\cdots +\gamma_{k}x_{i,k},$$

where *x*_*i*,0_, *x*_*i*,1_, … , *x*_*i*, *k*_ are the covariates of the model, with *x*_*i*,0_ being the intercept. The parameters *γ*_0_, *γ*_1_, … , *γ*_*k*_ are therefore the coefficients of the covariates to be estimated.

The parameter *δ* in Eq (4), which modifies the time trend of the Poisson intensity, is coherent with the Weibull hypothesis for the baseline intensity of the process [31][32], i.e. the part of the intensity function that does not depend on the covariates. Rewriting Eq (4) as

$$\lambda_{i}\left(t\right)=\exp\left(\gamma_{0}x_{i,0}\right)\delta t^{\delta -1}\exp\left(\gamma_{1}x_{i,1}+\cdots +\gamma_{k}x_{i,k}\right)$$ (6)

shows that the intensity in Eq (4) can be factorized as

$$\lambda_{i}\left(t\right)=\lambda_{i,0}\left(t\right)\lambda_{i,x}\left(t\right),$$

where the term λ_*i*,0_(*t*) = exp (*γ*_0_) *δt*^*δ*−1^ does not depend on the covariates (notice *x*_*i*,0_ = 1), while λ_*i*, *x*_ is the covariate-dependent part of the intensity.

The estimation of the models was performed using maximum likelihood. Given Eq (4), the log-likelihood of each model can be written as

$$\begin{array}{c} l\left(\delta ,\gamma \right)=N\log\delta +\left(\delta -1\right)\sum_{i=1}^{m}\sum_{j=1}^{n_{i}}\log t_{i,j}+\sum_{i=1}^{m}n_{i}x_{i}^{\prime }\gamma -\sum_{i=1}^{m}T^{\delta }\exp\left(x_{i}^{\prime }\gamma \right), \end{array}$$ (7)

where *n*_*i*_ is the number of claims for unit *i*, $N=\sum_{i=1}^{m}n_{i}$ is the total number of claims in the data set (*m* being the total number of units), *t*_*i*, *j*_ is the time in which claim *j* of unit *i* was reported, and *T* is the time length in years of the observation window, once we assume that January 1st 2004 is equal to the origin, i.e. time zero.

As we have mentioned, the model for the claims due to injuries and deaths at birth is different, since these claims can only arise from departments of obstetrics and gynecology. Hence the log-likelihood Eq (7) for such claims does not include the covariates *x*_*i*,3_, ⋯ , *x*_*i*,10_, so that the Eq (6) reduces to

$$\lambda_{i}\left(t\right)=\exp\left(\gamma_{0}x_{i,0}\right)\delta t^{\delta -1}\exp\left(\gamma_{1}x_{i,1}+\gamma_{2}x_{i,2}\right).$$

### A2.2—On models for the amounts

It is well known that if *Y* = log(*C*) follows a normal distribution with mean *μ* and variance *σ*^2^, then the expected value of *C* = exp(*Y*) is equal to $\exp(\mu +\frac{\sigma^{2}}{2})$. Also, because of monotonicity of the exponential function, the medians of *Y* and *C* are equal to *μ* and exp(*μ*), respectively.

We define the p-*th* quantile *y*_*p*_ of the log-cost by *P*(*Y* ≤ *y*_*p*_) = *p*. The corresponding cost quantile is therefore *q*_*p*_ = exp(*y*_*p*_) = exp(*μ* + *z*_*p*_ *σ*), where *z*_*p*_ is the p-*th* quantile of a standard Gaussian distribution.

If we set *p*_0_ = *P*(*C* = 0), it is not difficult to show that the overall p-*th* quantile of *C*, taking into account the point mass probability at zero, is *q*_*p*_ = exp((*μ* + *z*_*ϵ*_ *σ*), with *ϵ* = min(1, (1 − *p*)/(1 − *p*_0_)).

Both regression models have been developed to assess the statistical significance of the different regions, of the medical departments and of time (allowing for a possible quadratic effect of time on the two outcomes as well). It is worth underlining that the two (distinct) model selection processes will in general produce *different* sets of significant covariates, which we call **V**_1_ and **V**_2_, for the logistic regression component and for the lognormal regression component of the overall model, respectively. As a consequence, some care must be used to properly keep track of this fact in the later production of forecasts for the costs.

For the different combinations of covariates, let *β*_1_ and *β*_2_ be the parameter vectors of the two components of the model. With ${\hat{\beta }}_{1}$ and ${\hat{\beta }}_{1}$ we indicate their estimates.

Given ${\hat{\beta }}_{1}$ and ${\hat{\beta }}_{1}$, one can easily obtain an estimate of the probability that the amount corresponding to a claim is equal to zero as

$$\hat{P}\left(C=0;v_{1,0}\right)=\exp\left({\hat{\beta }}_{1}^{\prime }v_{1,0}\right)/\left(1+\exp\left({\hat{\beta }}_{1}^{\prime }v_{1,0}\right)\right).$$

Prediction intervals for such probability can also be readily obtained from the logistic regression analysis.

The estimated expected value of cost, for a value of the covariate vector **V**_2_ = **v**_2,0_, is

$$\hat{\mu }\left(v_{2,0}\right)=\hat{E}\left(C|C>0;v_{2,0}\right)=\exp\left({\hat{\beta }}_{2}^{\prime }v_{2,0}+\frac{{\hat{\sigma }}^{2}}{2}\right),$$

where $\hat{\sigma }$ is the variance estimate obtained from fitting the lognormal model. The estimation of expected values is notably difficult for the lognormal model, because of a problem of bias, and we recommend using predicted quantiles instead.

The estimated overall expected cost for **V**_1_ = **v**_1,0_ and **V**_2_ = **v**_2,0_ is

$$\begin{array}{c} \hat{E}\left(C;v_{1,0},v_{2,0}\right)=\hat{P}\left(C>0;v_{1,0}\right)\hat{E}\left(C|C>0;v_{2,0}\right). \end{array}$$ (8)

For ease of notation, in what follows, we drop **v**_1,0_ and **v**_2,0_, so that, for example, $\hat{E}(C;v_{1,0},v_{2,0})$ becomes $\hat{E}(C)$.

Clearly, it is possible to construct approximate *α* level prediction intervals $\left(\exp({\hat{\mu }}_{L}),\exp({\hat{\mu }}_{U})\right)$ for the conditional expected values *E*(*C*|*C* > 0). By keeping the variance estimate fixed, we get $\left(\exp(\hat{\mu }-z_{\alpha /2}{\hat{\sigma }}^{2}/2),\exp(\hat{\mu }+z_{\alpha /2}{\hat{\sigma }}^{2}/2)\right)$. It is relevant to note that fixing the variance generally underestimates the sampling variability of the predictions, even if, on the other side, it simplifies computations.

Prediction intervals for the *overall* mean costs can then be obtained by exploiting the independence between the sampling distributions of ${\hat{\beta }}_{1}$ and ${\hat{\beta }}_{2}$. By using $\alpha =\sqrt{0.95}=0.97468$ as the confidence level for the prediction intervals for the two terms in Eq (8), it is possible to show that 0.95 is a lower bound for the (approximate) confidence level for the prediction interval of *E*(*C*; **v**_1,0_, **v**_2,0_), constructed as $\left({\hat{p}}_{L}\exp({\hat{\mu }}_{L}+{\hat{\sigma }}^{2}/2),{\hat{p}}_{U}\exp({\hat{\mu }}_{U}+{\hat{\sigma }}^{2}/2)\right)$, where ${\hat{p}}_{L}$ and ${\hat{p}}_{U}$ are the lower and upper extremes of the *α* prediction interval for *P*(*C* > 0) = 1 − *P*(*C* = 0), as obtained from the logistic regression model.

Similarly, an approximate *α* level prediction interval for the *p*−th quantile *q*_*p*_ is obtained as $\left(\exp({\hat{\mu }}_{L}+\hat{\sigma }\;{\hat{\epsilon }}_{L}),\exp({\hat{\mu }}_{U}+\hat{\sigma }\;{\hat{\epsilon }}_{U})\right)$, where ${\hat{\epsilon }}_{L}=\min(1,\left(1-p\right)/{\hat{p}}_{L})$ and ${\hat{\epsilon }}_{U}=\min(1,\left(1-p\right)/{\hat{p}}_{U})$.

Last, let *N* be the number of events of a given kind in a given time interval, and *C*_1_, … , *C*_*N*_ their associated (*i.i.d.*) amounts. If one assumes that the expected values of the *C*_*i*_ do not depend on *N*, then the expected value of the overall amount for the time interval is

$$\begin{array}{c} E\;\left(\sum_{i=1}^{N}C_{i}\right)=E_{N}\;\left[E\;\left(\sum_{i=1}^{N}C_{i}|N\right)\right]=E_{N}\;\left[\sum_{i=1}^{N}E\left(C_{i}|N\right)\right]=E_{N}\left[N\;E\;\left(C\right)\right]=E\left(N\right)E\left(C\right), \end{array}$$

i.e. the product of the expected number of events and the expected amount for each event.

### A2.3—On tail costs

In this appendix we describe the predicted numbers of events (*N*), the probabilities that some of them lie in the extreme tail of the amount distribution, and the estimated Expected Shortfall (*ES*), or the average cost among costs greater than the (1 − *α*) − level quantile of the cost distribution.

Indeed, the cost quantiles are available, and the conditional distribution of the number *V* of extreme claims out of the *N* = *n* is a Binomial(*n*, *α*), where *α* is the tail area corresponding to the quantile amount *q*_1 − *α*_ of interest, as estimated from the analysis of the amounts. For example, the probability of observing *k* or more claims out of the *n* whose associated amounts are greater than or equal to *q*_1 − *α*_ is equal to

P(V≥k)=∑j=knnjαj1-αn-j,

where *α* = *P*(*C* ≥ *q*_1 − *α*_) = *P*(*C* ≥ *q*_1 − *α*_|*C* > 0)*P*(*C* > 0), and where all quantities are estimated from data. Note that this procedure is similar to the back-testing approach that is sometimes used in the verification of Value-at-Risk in risk management [7]. For large values of *n* (and as long as *nα*(1 − *α*) > 7, say) one may use the normal approximation to the binomial distribution and use the expression

$$P\left(V\geq k\right)\approx 1-\Phi \left(\frac{k-n\alpha }{\sqrt{n\alpha (1-\alpha )}}\right),$$

with Φ the standard normal cdf.

Let us now turn to the estimated expected amounts (or Expected Shortfall—*ES*—in risk management terminology), conditionally on the amount being positive (which happens with some probability 1 − *p*_0_), and in particular in the *α*-probability upper tail of the distribution. It is easy to check that

$$\begin{array}{c} E\left(C|C\geq q_{1-\alpha }\right)=e^{\mu +\frac{\sigma^{2}}{2}}\left[1_{\left\{\alpha \geq 1-p_{0}\right\}}+1_{\left\{\alpha <1-p_{0}\right\}}\frac{1-p_{0}}{\alpha }\Phi \left(\sigma -\Phi^{-1}\left(\frac{1-p_{0}-\alpha }{1-p_{0}}\right)\right)\right], \end{array}$$

where again we plug-in all the estimates to obtain such conditional expected values consistently. In our analyses we provide the quantity based on (*q*_0.90_) as an example (hence choosing *α* = 0.10).

## Appendix 3—Final models for amounts

In this appendix we report the details of the final models selected for the amounts associated to injuries.

Details of the final model selected for the probability that cost is equal to zero are shown in Table 18. That model has identified statistically significant effects for several regions, medical departments, and for calendar time (quadratic effect). For the model for (positive) cost, the model selection process identified significant effects for the Sicilia and Veneto regions, as well as for all medical departments and for time. The estimated parameter values are shown in Table 19.

**Table 18:** Final model for the probability that Cost (payment) is equal to zero for claims given by injuries.

	Estimate	Std.Error	z-value	P-value	Sign.
(Intercept)	0.866	0.0928	9.335	2e-16	#
Lazio	-0.7412	0.1297	-5.716	1.09e-08	#
Liguria	-1.1214	0.0782	-14.349	2e-16	#
Lombardia	-1.4918	0.0708	-21.067	2e-16	#
Marche	-0.7558	0.1253	-6.03	1.64e-09	#
Nordest	-0.5718	0.0840	-6.81	9.75e-12	#
Sicilia	-1.1656	0.1583	-7.362	1.81e-13	#
Toscana	-0.7137	0.1051	-6.791	1.11e-11	#
Valle D’Aosta	-0.4819	0.1959	-2.46	0.0139	*
Veneto	-1.3675	0.1342	-10.188	2e-16	#
Anesthesia (AN)	-0.9556	0.1245	-7.678	1.62e-14	#
Surgery (SU)	-0.6211	0.0540	-11.502	2e-16	#
Orthopedics (OR)	-0.9493	0.0673	-14.1	2e-16	#
Obstetrics and Gynecology (GY)	-0.6007	0.0984	-6.105	1.03e-09	#
Health Support Services (HS)	-0.2771	0.1009	-2.745	0.0061	+
Emergency (ED)	-0.4210	0.0665	-6.329	2.48e-10	#
time	0.1958	0.0388	5.053	4.35e-07	#
time^2^	-0.0369	0.0051	-7.188	6.57e-13	#

Significance (Sign.) codes: 0 ≤ # ≤ 0.001 ≤ + ≤ 0.01 ≤ * ≤ 0.05

**Table 19:** Final model for Cost (payment), conditionally on its being greater than zero, for claims given by injuries.

	Estimate	Std.Error	z-value	P-value	Sign.
(Intercept)	8.6872	0.0788	110.274	2e-16	#
Sicilia	-0.6021	0.1432	-4.204	2.65e-05	#
Veneto	-0.6840	0.1065	-6.421	1.44e-10	#
Anesthesia (AN)	-0.7346	0.1130	-6.499	8.67e-11	#
Surgery (SU)	0.7986	0.0721	11.08	2e-16	#
General Medicine (ME)	0.3727	0.0919	4.057	5.03e-05	#
Missing Information (NA)	0.6323	0.1315	4.81	1.54e-06	#
Orthopedics (OR)	1.0137	0.0771	13.154	2e-16	#
Obstetrics and Gynecology (GY)	1.0293	0.1003	10.259	2e-16	#
Not Classifiable (NC)	-0.3554	0.0890	-3.992	6.63e-05	#
Emergency (ED)	0.1813	0.0812	2.234	0.0256	*
time	0.0763	0.0347	2.2	0.0279	*
time^2^	-0.0170	0.0045	-3.785	0.0002	#

Significance (Sign.) codes: 0 ≤ # ≤ 0.001 ≤ + ≤ 0.01 ≤ * ≤ 0.05

## Supporting Information

S1 File S1_File.zip. The data for the largest region in the data set (Lombardia/Lombardy) are freely available for download. For each claim, we provide: code of the hospital (Hospital), department (Department), time of the event (Time), year (Year), type of claim (Event), CMI (CMI), number of patients for the hospital in 2012 (Patients 2012), reserved amounts (Reserves) and final amounts (Final Amount). The names of the Brokers have been anonymized: 1BR23 indicates Hospital 23 of Broker 1BR. Time has been rescaled, so that Day 1 corresponds to January 1 2004.
(ZIP)
